# Investigating the Relation between Stochastic Differentiation, Homeostasis and Clonal Expansion in Intestinal Crypts via Multiscale Modeling

**DOI:** 10.1371/journal.pone.0097272

**Published:** 2014-05-28

**Authors:** Alex Graudenzi, Giulio Caravagna, Giovanni De Matteis, Marco Antoniotti

**Affiliations:** 1 Dept. of Informatics, Systems and Communication, University of Milan-Bicocca, Milan, Italy; 2 Department of Mathematics and Information Sciences, Northumbria University, Newcastle, United Kingdom; Universitat Pompeu Fabra, Spain

## Abstract

Colorectal tumors originate and develop within intestinal crypts. Even though some of the essential phenomena that characterize crypt structure and dynamics have been effectively described in the past, the relation between the differentiation process and the overall crypt homeostasis is still only partially understood. We here investigate this relation and other important biological phenomena by introducing a novel multiscale model that combines a morphological description of the crypt with a gene regulation model: the emergent dynamical behavior of the underlying gene regulatory network drives cell growth and differentiation processes, linking the two distinct spatio-temporal levels. The model relies on a few a priori assumptions, yet accounting for several key processes related to crypt functioning, such as: dynamic gene activation patterns, stochastic differentiation, signaling pathways ruling cell adhesion properties, cell displacement, cell growth, mitosis, apoptosis and the presence of biological noise. We show that this modeling approach captures the major dynamical phenomena that characterize the regular physiology of crypts, such as cell sorting, coordinate migration, dynamic turnover, stem cell niche correct positioning and clonal expansion. All in all, the model suggests that the process of stochastic differentiation might be sufficient to drive the crypt to homeostasis, under certain crypt configurations. Besides, our approach allows to make precise quantitative inferences that, when possible, were matched to the current biological knowledge and it permits to investigate the role of gene-level perturbations, with reference to cancer development. We also remark the theoretical framework is general and may be applied to different tissues, organs or organisms.

## Introduction

Intestinal crypts are invaginations in the intestine connective tissue, which are the *loci* where *colorectal tumors*, one of the major causes of deaths in adults, originate and develop [1]–[4]. These particular structures have been quite precisely characterized, highlighting a fast renewing single layer epithelium in which distinct cell populations are rather sharply stratified and cells coordinately migrate from the *multi-potent stem cell* niche (at bottom) toward the intestinal lumen, with some exceptions [5]–[9]. As long as cells move upward they divide and differentiate through intermediated stages, according to a hypothesized *lineage commitment tree*, which ensures the correct functioning of the crypt and its resistance to perturbations and biological noise. The complex interplay between *cell proliferation*, *differentiation*, *migration* and *apoptosis* results in the overall homeostasis of the system. Chemical gradients ruled by key signaling pathways such as *Wnt*, *Notch*, *Eph/ephrin* have a crucial role in all these processes and, when progressively mutated or altered, cancerous structures may emerge [10], [11].

Mathematical and computational models have been widely used to describe intestinal crypts (see [12], [13] and references therein). Among these, *compartmental models* analyze population dynamics via mean-field approaches without accounting for the spatial and mechanical properties of the crypts [14], [15]. In order to consider *space*, both *in-lattice* and *off-lattice* models have been defined. The former use simplified cellular automata-based representations of crypts to account for cell displacement, movement and interactions (see, e.g., [16], [17]). The latter strive to model more directly the geometry and the physics of crypts, but, as they involve bio-mechanical forces and complex geometries (e.g., *Voronoi diagrams*), the spaces of parameters and variables dramatically enlarges (see, e.g., [18]–[20]). As usual, the best trade-off between the complexity of the model and that of the modeled phenomena depends on the aim of the research.

Even if a large list of important phenomena, such as the spatial arrangement of cell population or the stem cell niche maintenance, have been described with noteworthy results with currently existing models, the relation between the underlying differentiation processes and the overall crypt homeostasis is still only partially understood. To investigate *in-silico* this relation and other important biological properties we here introduce a *novel* multiscale model of intestinal crypt dynamics, presented in a preliminary version in [21]. The *multiscale* approach allows to consider, at different abstraction levels, phenomena happening at distinct spatiotemporal scales, as well as the hierarchy and the communication rules among them [22], [23]. In the case of crypts, these include intra-cellular processes such as gene regulation and intra-cellular communication, and inter-cellular processes such as signaling pathways, inter-cellular communication and microenvironment interactions. Their joint complex interaction allows to quantify, at the level of *tissues*, some key properties of crypts such as their spatial patterning, cellular migration, overall homeostasis and clonal expansion.

The foundations of our model lay in *statistical physics* and in *complex systems theory*, as the main rationale is to use the simplest possible model to reproduce relevant complex phenomena, also allowing for a comparison with experimental data and biological knowledge [24]. Thus, our model relies on few *a priori* assumptions and constraints, and most of its properties are *emergent*. The model is composed of two distinct levels, accounting for the crypt *morphology* and the underlying cellular *Gene Regulatory Network* (GRN).

Crypt morphology, the spatial level of the model, is described via the well-known *in-lattice Cellular Potts Model* (CPM), already proven to reproduce several properties of real systems [25]–[27]. In this discrete representation cells are represented as contiguous lattice sites (i.e. *pixels*), and their movement (via pixel re-assignment) is driven by an energy minimization criterion accounting for cellular type, position, age and size. Despite being a simplification of the real crypt morphology, important biological aspects such as cell heterogeneity and noise are effectively accounted for with this approach.

GRNs are modeled as *Noisy Random Boolean Networks* (NRBNs, [28], [29]), a simplified model of gene regulation that allows to relate the processes of cell differentiation with the robustness of cells against biological noise and perturbations [30]. This widely used model considers genes as a “black box” and accounts for simplified regulatory interactions, i.e., by not considering explicitly the biochemical details of entities and relations, while focusing on the *emergent dynamical behavior* of networks in terms of *gene activation patterns* that characterize the cellular activity. Following an approach typical of complex systems, the aim is to investigate the so called *generic* (or universal) *properties* and *principles* of biological systems, i.e., those properties that are shared by a broad range of distinct systems, in this case by gene regulatory networks. A powerful instrument in this regard is the statistical analysis of *ensembles* of randomly simulated networks with certain biological constraints, in order to scan the huge space in which real networks (on which the information is still missing) are likely to be found. Even though the Boolean modeling approach relies on drastic simplifications, it was repeatedly proven fruitful in investigating the generic properties of generally large networks, without the need of using the high number of (usually not available) parameters necessary in other approaches, e.g. modeling via differential evolution equations. In fact, classical RBNs were efficiently used to surrogate GRN models until complete information on real networks started to become available [31]–[36]. Moreover, the simulation of the dynamics of (usually small) biologically plausible Boolean networks recently gained attention, starting from specific works on regulatory circuits [37]–[39]. We place our model closer to the large-networks approach, with the current goal of investigating the generic properties of gene networks, yet with the explicit future objective of approaching the modeling of more biologically realistic architectures, given the generality of the cell differentiation model here introduced.

Along the lines of [30], each cell type is characterized by particular patterns, whose stability with respect to biological noise is related to its degree of differentiation [40]–[43]. The approach is general (i.e. it is not related to a specific organism) and is able to reproduce key phenomena of the differentiation processes such as: 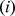 *hierarchical differentiation*, i.e. from toti-/multi-potent stem cells to fully differentiated cells through intermediate stages; 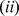 *stochastic differentiation*, i.e. a stochastic process rules certain fate decisions and directions; 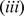 *deterministic differentiation*, i.e. specific signals or mutations trigger certain differentiation fates; 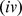 *induced pluripotency*, i.e. fully differentiated cells can return to a pluripotent stage through the perturbation of some key genes [44].

In our multiscale approach, the GRN dynamics drives cellular growth and the differentiation fate of cells, thus linking the GRN to the crypt morphology.

Following the work by Wong *et al.* [17], in this paper we investigate key dynamical properties of crypts and, in particular, we show that the stochastic differentiation process is itself sufficient to ensure the crypt homeostasis, under certain conditions. Our novel approach permits to relate the genotype-level model of GRN to complex phenotypes and quantitative measures of crucial phenomena occurring in crypts, such as: 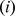 the spontaneous sorting and segregation of cell populations in different compartments, driven by cell adhesion processes; 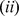 the maintenance of the correct proportion between cell populations with distinct functions in the crypt; 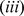 the fast renewal process of cells, as resulting from the interplay involving newborn cells and dead ones (either because expulsion in the lumen or apoptosis, which should be modeled per se, cfr. [45]); 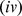 the coordinate migration of cells from the stem cell-niche toward the intestinal lumen at the top of the crypt; 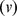 the noise-driven progressive differentiation of totipotent stem cells in 8 hierarchical cell types, through transit amplifying stages; 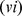 the clonal expansion of sub-populations deriving from single progenitors.

Despite the affinity with [17], our work contains several major differences (see the paragraph in the Model section for a detailed comparison). For instance, our GRN-based differentiation model consists of a stochastic fate decision process depending on an *emergent* lineage tree and, also, the key features of the cell cycle that we consider emerge by the dynamical properties of the underlying GRNs, while in [17] are superimposed. In addition, one of the major motivations for using a multiscale model is the possibility of explicitly perturbing the GRN, simulating different kinds of mutations and alterations at the genome level, which we leave as future work. In this way, one can analyze the influence of the progressive accumulation of genetic alterations on the overall dynamical tissue-level behavior of crypts, thus providing a powerful instrument to investigate the possible emergence of aberrant structures such as colorectal cancer.

In this regard, the *dynamical* characterization of genotypic and phenotypic phenomena recently gained greater attention [46], [47]. For example, in [48] cancer development is depicted as a dynamical process characterized by metastable states (i.e. *attractors* in the terminology of *dynamical systems*) in which stochastic transitions account for cancer heterogeneity and phenotypic equilibria. In general, a dynamical approach provides more information than the static counterpart, given the inherently *evolutionary* nature of cancer. In this respect our model is, to the best of our knowledge, the first attempt to combine a dynamical attractor-based model of GRN with a morphological multicellular model, allowing for innovative analysis perspectives.

Besides, our model is conceived to be flexible and modular, thus both its spatial and gene-level components may be refined to include, for instance, signaling pathways and chemical gradients. We also remark that our modeling approach is general and, in principle, can be applied to any kind of tissue, organ or organism.

The paper is structured as follows. A brief overview of the biology of the crypts is given in the next section. Next, the internal and external components of the model are described, as well as their multiscale link. The results of the analyses on the model are discussed in the subsequent section. Finally, conclusions are drawn.

### A brief overview of the biology of the intestine

Among many, the main functions of the human intestine are 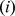 *food digestion* and 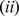 *nutrients absorption*, while several other minor processes are linked to the general homeostasis of the system and to the immune system mechanisms. The distinct compartments of the intestine are composed by muscular, stromal and cuboidal epithelial cell. The lining of the small intestine is composed by a single-layer epithelium that covers the villi and the crypts of Lieberkühn, which are the object of our model. Notice that in the large intestine there are no villi, but only crypts (see [1], [49] and references therein).

Four distinct differentiated epithelial cell types are present in the crypt, all descending from multi-potent *stem cells*, which give rise to a progeny that undergoes a post-mitotic progressive differentiation process, characterized by the presence of partially differentiated cells in *transit amplifying stages* (see [50] for an exhaustive discussion). In particular, the four epithelial fully differentiated lineages are: *enterocytes*, performing both absorptive and digestive activity via hydrolases secretion, *Goblet cell*, secreting mucus to protect the absorptive cells from digestion, *Paneth cell*, performing defensive tasks by means of antimicrobial peptides and enzymes and *enteroendocrine* cells (a general category with 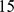 subtypes) entailed in many different tasks and signaling pathways [49], [51], [52]. Other minor cell types, such as M-cells and Brush cells have been also detected [53].

**Figure 1:**
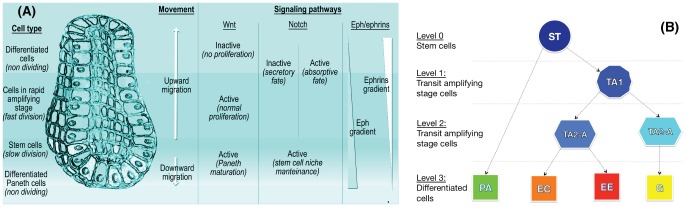
Crypt morphology and differentiation tree. (**A**) A depiction of the crypt morphology, with the direction of cell migration and a schematized representation of the interplay among the key signaling pathways (taken from [13]). All cells but stem and Paneth migrate upward. The three major signaling pathways involved in the crypt activity are the Wnt, the Notch and the Eph/ephrins pathways. In (**B**) the crypt differentiation tree is shown, involving stem (St), transit amplifying stage (TA1, TA2-A, TA2-B), Paneth (Pa), Goblet (Go), enteroendocrine (Ee) and enterocyte (Ec) cells.

Cell type populations are segregated in distinct portions of the crypt: the proliferative cell compartments is in the lower part of the crypt, all other types but Paneth cells reside at its top. Stem cells are sited at the bottom of the crypt in a specific niche, intermingled or just above Paneth cells, according to different hypotheses [54] (see Figure 1). The overall dynamics is a coordinated upward migration of enterocyte, Goblet and enteroendocrine cells from the stem-cell niche [55]. At the end of migration these cells are shed into the intestinal lumen; this loss of cells balances the production from the base of crypt. Paneth cells are the only cells that move downward and reside at the bottom of the crypt (see [2], [50] and references therein). In this complex coordinate movement cell populations maintain the segregation in distinct compartments [1].

The cellular turnover is fast. For instance, in mice the crypt progenitors divide every 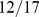 hours, so around 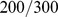 cells per day are generated, and they successively undergo up to five rounds of cell division while migrating upwards [49], [56]. Accordingly, migrating cells move from the base to the surface in about 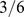 days, while Paneth cells, which live for about 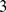 to 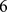 weeks, and stem cells localize at the crypt bottom and escape this flow [2], [57].

The signaling pathways throughout the epithelial cells and between the epithelium and the mesenchyme are fundamental for many phenomena such as spatial patterning, proliferation in transit-amplifying compartments, commitment to specific lineages, differentiation and apoptosis [49]. We briefly describe the three most important signaling pathways involved in these processes.

The *Wnt* pathway is supposed to drive cell proliferation and to rule the differentiation fate. Also, it is responsible of avoiding the immediate differentiation, and activates the expression of the Notch pathway [49]. The activation of this pathway keeps the crypts in a normal proliferative state, whereas its inactivation stops the division/differentiation process. In [54], [58] it is shown that its correct activation is required to determine the Paneth cell fate and lineage.

The *Notch* pathway is involved in the control of the spatial patterning and the cell fate commitment, with the task of ensuring the status of undifferentiated proliferative cells in the progenitors compartment, in a concerted combination with the Wnt pathway [59]. This signaling pathway mediates also *lateral inhibition*, which forces the cells to diversify: some cells express Notch ligands and activate the Notch signaling in the neighbors, while avoiding their own activation. In this way they commit to the finally differentiated fate. In the other cells the Notch ligands are inhibited while the Notch pathway is active within the cell itself; in this way they maintain the possibility of differentiating in any possible way. Multi-potent crypt progenitors are supposed to be maintained only when both Wnt and Notch pathways are active [53].

Finally, the interaction between *Eph* receptors and *ephrin* ligands can trigger a downstream cascade that controls cell-cell adhesion, cell-substrate adhesion, cytoskeletal organization and cell-extracellular matrix binding, influencing the formation and the stability of tight, adherence and gap junctions and integrin functions [60]–[63].

## Methods: A Multiscale Model of Intestinal Crypts Dynamics

We separately introduce all the model components with respect to the key biological processes we account for. A detailed mathematical definition of the model and of the simulation algorithms can be found in File S1.

### Crypt morphology as a collective multi-cellular dynamical structure

**Figure 2:**
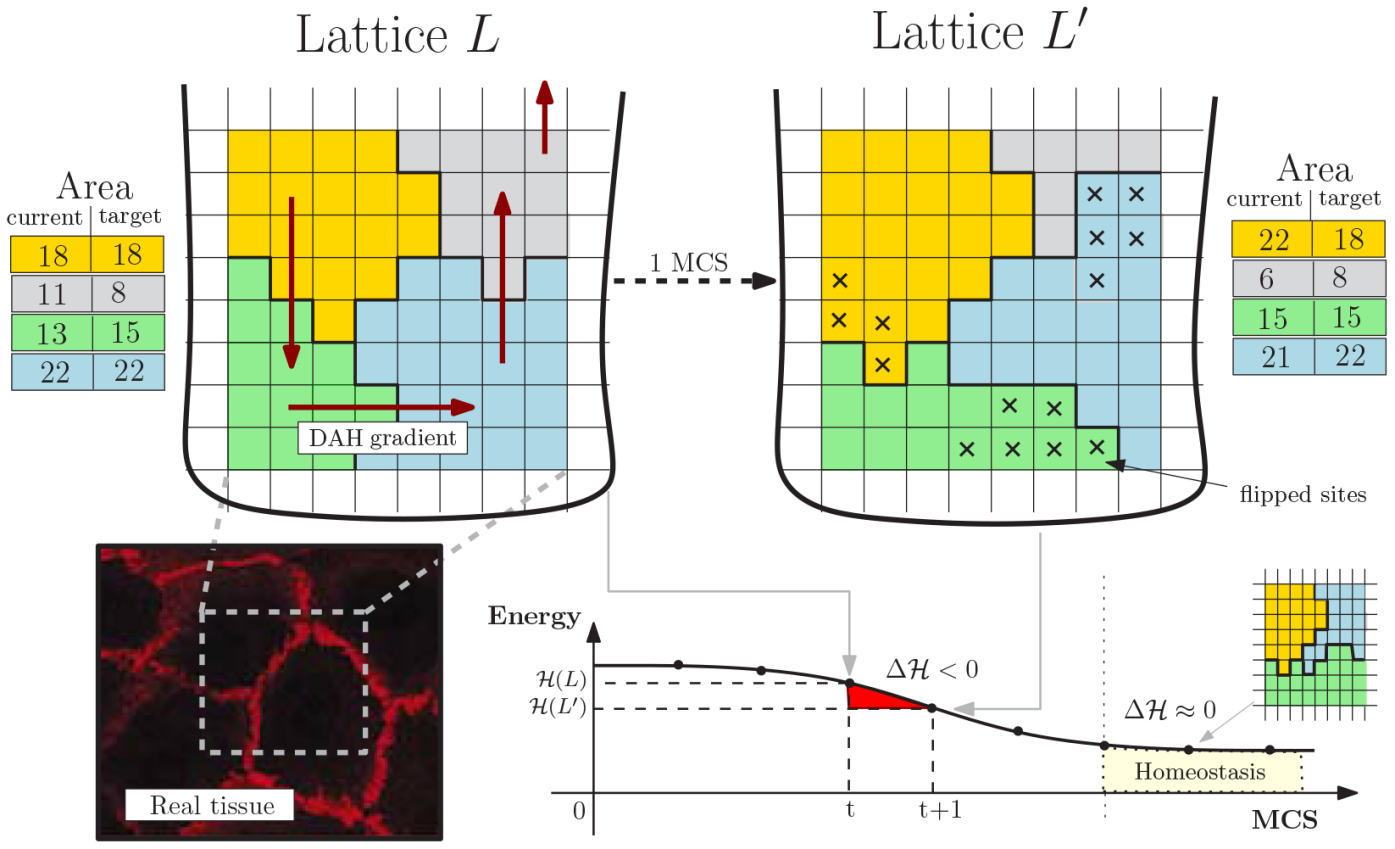
Cellular Potts Model. Lattice-based representation of the crypt tissue as a opened and rolled out lattice 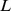, with 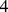 cells. The energy gradient induced by the DAH via 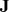 and the current/target area for each cell are represented. An example MCS step is shown resulting in the re-arrangement of 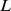 in favor of 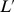 (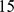 flips accepted), whose hamiltonian energy is lower. The final tissue stratification is achieved when 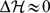 where the grey cell is expelled in the lumen. In the left corner an example picture of real tissue is displayed.

We adopt a simple geometrical representation of crypts inspired by the theory of cellular automata and statistical physics: the *Cellular Potts Model* (CPM, [25]), often used to account for energy-driven spatial patterns formation [27]. A graphical representation of the CPM model is shown in Figure 2.

We display cells over a rigid 2D grid by assuming a (simplified) perfectly cylindrical crypt, opened and rolled out onto a rectangular 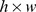 lattice 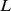 through periodic boundary conditions. Each cell is delimited by connected domains as in cellular automata so a cell 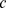, denoted as 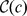, consists of all lattice sites of 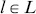 with value 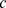, that is
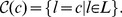
(1)For each disposition of cells a *energy level* is evaluated via a Potts-like Hamiltonian function 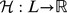 accounting for the energy required for each mutual interaction and other physical quantities (see below). A discrete-time stochastic process of cellular re-arrangement drives the lattice to configurations minimizing the overall hamiltonian energy. The time unit of these steps is the so-called *Monte-Carlo Step* (MCS). The operation key to cellular re-arrangement is that of flipping a lattice site of a cell in favor of another cell, thus modeling cellular movement over the lattice. The changes in the lattice which can happen in a single MCS are sketched as:

1. let 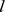 be a lattice site selected with uniform probability in 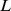, let 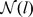 be its set of neighbor sites, select a random 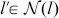;
2. assign site 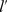 to the cell in 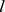 with probability
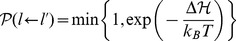
(2)where 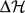 is the gain of energy (i.e., the hamiltonian difference) in accepting the flip;
3. repeat steps 1–2 for 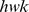 times, with 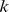 a positive integer.

In step 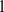 we set 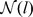 to the standard Von Neumann neighborhood: if 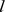 is in position 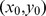 its neighbors of degree 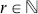 are 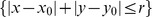. Step 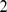 is the probabilistic re-arrangement of a single lattice site; by iterating 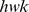 times a single MCS is simulated and the new lattice configuration displays the cells which moved in that time unit. The Boltzmann distribution is used in equation (2) to drive cells to the configuration with minimum energy; such a distribution depends on the temperature 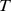 and on the Boltzmann constant 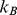 (the factor 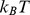 gives account of the amplitude of the cell membrane fluctuations at boundaries).

Cell sorting is the phenomenon by which population of cells of distinct type segregate and form distinct compartments or different tissues. According to Steinberg's *Differential Adhesion Hypothesis* (DAH, [64]), cell sorting may be due to cell motility combined with differences in intercellular adhesiveness and these phenomena in crypts are clearly related to the functioning of the Eph/ephrins signaling pathway (see the Biological background section). In detail, under DAH tissues are considered as vascoelastic liquids whose tissue surface tension can be measured. These tensions correspond to the mutual cellular behavior thought to be responsible for the formation of complex multi-cellular structures. In our model we adopt a *thermodynamical* interpretation of Steinberg's hypothesis to account for the effects of cell adhesion molecules in a simple way. Along the lines of [17] we assume that a certain amount of energy is required to keep two cells tied to each other, and we assume that higher energy is required to stick together cells of distinct types. Since the surface tensions can be determined for various tissues, we can use realistic parameter values for these energies [26], [65], [66]. In this way, we implicitly include in our model an abstraction of one of the most important signaling pathways involved in the phenomena relevant to crypt homeostasis.

Therefore, the energy minimized by equation (2) is given by the hamiltonian function
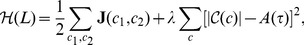
(3)where 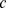 denotes a generic cell of type 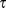 and 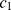 and 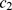 are different neighbor cells. Function 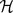 accounts for:

- the amount of energy 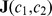 required to stick tied 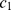 and 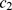, according to the DAH;
- the tendency of each cell of type 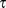 to grow towards some target area 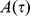.

Thus, the target lattice configuration the system is driven to is that where the amount of bond energy is minimal and cells tend to grow up to their target size. Notice that the the total area of a cell is measured as the total number of pixels currently occupied by the cell, i.e., 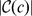, and the capacity to deform a cell membrane is given by the size constraint 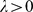. As far as the DAH is concerned, 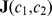 is the surface energy between the two cells (defined on the basis of the gradients of Eph receptors and ephrin ligands), and is defined according to their cell type (see Tables 1 and 2).

**Table 1:** Parameters of the Noisy Random Boolean Networks modeling the Gene Regulatory Network of intestinal crypts, and of the Cellular Potts model of crypt morphology.

Noisy Random Boolean Networks			
*Symbol*	*Value*	*Description*	*Source*
N	100	number of GRN genes (NRBN nodes)	*estimation in accordance with the driver genes for colorectal cancer * [*85*] ^*^
|*K*|	3	average GRN connectivity	*input lineage tree* ^*^
-	*scale-free*	GRN topology	[83]
*γ*	2.3	Power-law exponent (scale-free GRNs)	[84]
-	*canalyzing*	type of boolean functions	[87]

Parameters with symbol^*^ are fit.

**Table 2:** Parameters of cellular adhesion (matrix **J**) for the cell types considered.

J	St	TA1	TA2-A	TA2-B	Pa	Go	Ec	Ee
St	2	−	−	−	−	−	−	*-*
TA1	12	5	−	−	−	−	−	*-*
TA2-A	35	30	15	−	−	−	−	*-*
TA2-B	35	30	15	15	−	−	−	*-*
Pa	8	20	40	40	2	−	−	*-*
Go	45	40	30	30	50	5	−	*-*
Ec	45	40	30	30	50	5	5	*-*
Ee	45	40	30	30	50	5	5	5

Furthermore, since crypts are not isolated systems, we both consider the expulsion of cells in the intestinal lumen (shedding of fully differentiated cells by mitotic pressure) and the presence of the *Extra Cellular Matrix* (ECM), i.e. the stroma scaffold surrounding crypts. Cell expulsion, which allows the renewal of cells in the crypt, is achieved by the migration of cells towards the top of lattice which, we recall, it is open. The ECM is modeled as a special cell type with un-constrained area (see File S1 for a detailed definition of function 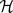 with the ECM cell type).

Finally, cells moving on a lattice eventually complete their cell-cycle. In our case mitosis follows cycle completion and a cell divides into two daughter cells, which are characterized by specific target areas. In particular, stem cells divide asymmetrically, producing a unique daughter (and the stem cell itself), whereas the other proliferative cells divide in two daughters that change type by following the differentiation fate ruled by the GRN dynamics.

### Noise-induced stochastic cellular differentiation via GRNs

We consider the 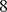 cell types 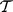 = {St, TA1, TA2–A, TA2–B, Pa, Go, Ec, Ee} shown in Figure 1, and we adopt the hypothesis that *more differentiated cells are more robust against biological noise*, because of more refined control mechanism against perturbations and random fluctuations. Accordingly, the toti-/multi-potent stem cell type is less robust against noise and is thus able to differentiate in any other cell type. In this regard, a wide literature is currently available on: 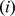 the role of noise in gene regulation, e.g., [43], [67]–[71], 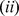 the relation between noise and the differentiation processes, e.g., [40], [72]–[76], 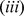 the hypothesis according to which the level of noise in undifferentiated cells is relatively higher, e.g., [41], [42], [77].

By using this intuitive idea we link noise-resistance to the *stochastic cellular differentiation* process, at the level of the GRN shared by all the cells in the crypt: once a cell divides, the specific cell type of its progeny depends on a random process, according to the underlying lineage commitment tree. In this paper, we adopt a simplified representation of such a GRN based on the *Random Boolean Networks* (RBNs, [31], [78], [79]) approach where genes, and the encoded proteins, are represented in a abstract “on”/“off” fashion. Despite the underlying abstractions, this model has proven fruitful in reproducing several key generic properties of real networks (see, e.g., [32], [34]–[36]). Intuitively, each gene is associated to a *boolean variable* 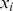: 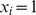, the “on” state, models the activation of the gene (i.e., production of a specific protein or RNA), conversely 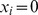 models the inactive gene. The interaction among the genes is represented via a *directed graph* where nodes are the binary variables, edges symbolize the regulation paths and each gene affects the neighbor genes via a boolean updating function 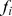 associated to each node.

The network graph represents the possible genetic interactions and is used to “simulate” the evolution of the GRN in a discrete-time, synchronously and deterministically. Let 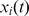 be the state of each gene 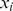 at time 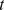, the new value of 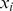 at time 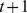 is a function of its connected components 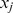, 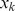, …, that is
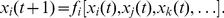
(4)


Given that the dynamics is synchronous and deterministic, *gene activation patterns* will eventually emerge from it; technically, these *RBN attractors* are stable *limit cycles* representing sequences of activations/inhibitions of genes, repeating in time [78]. Patterns will be used as a compact representation of the underlying GRN, and their *stability* will be used to model the noise-resistance of each cellular type [28].

This is the so-called *Noisy Random Boolean Networks* (NRBNs, [29], [30]) model of regulatory network. Together with the DAH-based adhesion energy matrix, this model of regulatory network implicitly includes within the multiscale model the relevant signaling pathways, as their influence is encoded in the various gene regulatory circuits, which, in turn, rule the overall crypt dynamics. We here remark that each cell of the system is characterized by the *same* NRBN, like all the cells of an organism share the same genome (i.e. GRN). The differences in the activity of the distinct cells is due to the particular dynamics of their own gene activation pattern (for instance, distinct cells of the same type own the same NRBN and the same gene activation pattern, but can be in different phases of the pattern).

**Figure 3:**
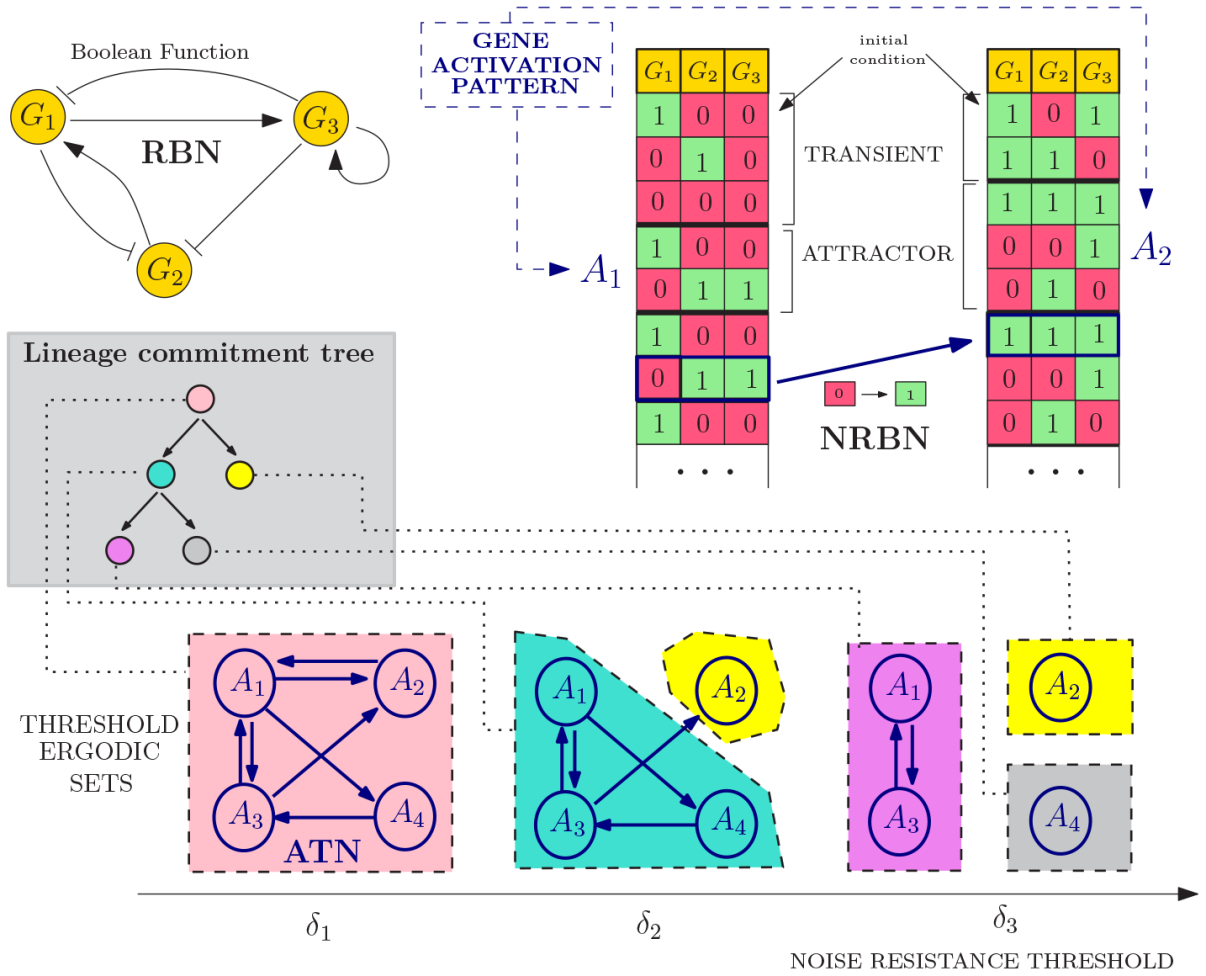
Noise-induced stochastic differentiation. An example NRBN with 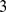 genes is shown, boolean functions are omitted. Two initial genetic configurations yield two gene activation patterns: attractors 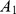 and 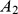, whose noise-resistance is evaluated via flipping different nodes in different phases and leading to an Attractors Transition Matrix. The emerging lineage commitment tree consists of 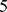 cell types (one for each Threshold Ergodic Set for the 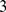 noise thresholds 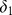, 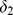 and 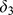). The differentiation level corresponds to the noise-resistance, e.g., the toti-/multi-potent stem-alike cell type (pink) roams among all possible gene activation patterns, the grey/yellow cell types are fully differentiated cells. This model of differentiation has branches, i.e. a newborn pink cell has probability of differentiating in a green or yellow cell proportional to the properties of the attractors (see Figure 4).

We sketch here its usage, which is schematized in Figure 3; for a exhaustive mathematical definition of NRBNs we refer to File S1. The process is as follows:

1. a random RBN is generated with some specific bio-inspired constraints (see below);
2. a set of GRN configurations representing the initial conditions of the RBN is generated by turning “on”/“off” the genes (i.e., assigning 0/1 values to all the variables 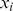);
3. for each configuration the dynamical trajectory of the GRN is generated via equation (4) (right table, Figure 3);
4. all the stable limit cycles of a GRN define its gene activation patterns (e.g., the attractors 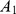 and 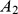 in Figure 3);
5. the stability to noise of each gene activation pattern is tested by performing random perturbations on each gene (i.e., temporary *flips*). A stable pattern is robust when the dynamical trajectory that follows a perturbations returns to the pattern itself. Notice that unstable patterns may determine new attractors;
6. by repeatedly performing step 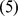, the stability of each gene activation pattern is numerically evaluated, determining the noise-induced probability of switching between patterns. The *Attractor Transition Network* (ATN, [30]) accounts for the relative probabilities of switching among patterns (see Figure 3);
7. the *connected components* of the transition network are *noise-driven connected gene activation patterns* used to define the *hierarchical differentiation tree* in Figure 1, more precisely:
  - *toti-/multi-potent stem cells* are the connected component of the ATN involving all the possible genetic patterns, through which the GRN continues to wander due to biological noise and random fluctuations;
  - according to the hypothesis that more differentiated cells are characterized by a higher resistance to noise, we define *threshold-dependent Attractor Transition Network* by pruning the probabilities below distinct thresholds, hence neglecting the transitions that are unlikely to occur in the lifetime of a cell: higher thresholds correspond to a better resistance against noise.

By performing this step recursively, we detect connected components of patterns in the transition network according to increasingly larger thresholds, termed *Threshold Ergodic Sets* (TESs) in the NRBN jargon, which are hierarchically assigned to the sub-types in the tree, according to the strategy defined in [30] (see bottom of Figure 3).

Larger thresholds progressively determine smaller and more fragmented ergodic sets, which correspond to more differentiated cell types. These sets reflect the usual assumptions that less differentiated cells, e.g., stem cells, can roam in the wider portion of the space of plausible genetic configurations for a cell (i.e., 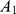, 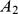, 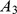 and 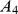 in Figure 3) and vice versa [71].

When all these steps are complete, the emerging hierarchy between the cell types is matched against the crypt differentiation tree of Figure 1, as sketched in Figure 3. If it matches, the generated NRBN is a network whose emergent cellular types are able to characterize the crypt lineage commitment tree and can be used in the morphological simulation. If it does not match, the NRBN is rejected and the process re-starts.

This strategy requires only a few *a priori* structural assumptions on the underlying GRN, along the usual *ensemble* approach to complex systems [31]. This makes sense since, in this case, it is undoubtedly difficult and hazardous to conjecture a specific human GRN. Instead, we aim at studying the general emergent properties of a class of networks and relating them to the crypt dynamics. In this respect, we generate NRBNs satisfying the structural constraints given by the current biological knowledge of real GRNs and select the *suitable* ones on the basis of their emergent dynamical behavior (see the Results section). Notice that, in line with the fact that the human GRN is unique, we should not expect to find many “suitable” networks.

### A multiscale link between GRNs and the morphology of the crypt

Each cell on the spatial model incorporates a specific GRN, which is characterized by specific gene activation patterns, related to the degree of differentiation. Three major cellular processes are then ruled by the internal NRBN dynamics, thus providing the link between GRNs and the morphological model: 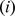 the length of the cell cycle, proportional to the weighted length of the gene activation patterns of each specific cell type, 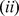 the cell growth rate, assumed to be linear in time, and 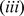 the differentiation process, as explained in the previous section. We remark that, without accounting explicitly for GRNs, 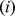 and 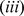 could not be emergent properties but should be assumed.

**Figure 4:**
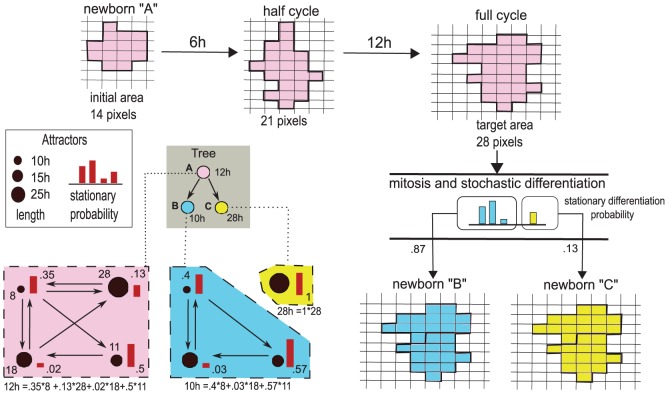
Multiscale link. Example representation of the multiscale link for three distinct cell types 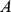 (pink), 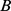 (blue) and 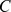 (yellow), belonging to the shown lineage tree. A specific Treshold Ergodic Set is associated to each cell type. The length of each attractor composing the TES is given by the size of the different circles, whereas the stationary distribution is represented by the red bars. The length of the cell cycles is then computed with Eq. 5. A cell cycle for a cell of type 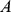 is shown in the upper row: the newborn cell starts with an initial area of 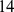 pixels and doubles its area in 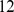 hours. At the end of its cycle it undergoes mitosis and differentiates stochastically. The stationary probabilities suggest that most likely daughter cells will be of type 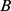, rather than 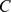. In the two scenarios the newborn cells will have different cell cycle length and division pace, and will lead to different differentiation fates. This shows how the GRN dynamics affects the tissue-level cell dynamics.

For clarity, in Figure 4 we represent the multiscale link and its effect on a growing cell.

#### Cell cycle length and time-scales conversion

Ergodic sets in the terminology of [30] are analogous to *ergodic* discrete-time Markov Chains, which are known to possess a *unique* computable stationary probability 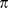 (see File S1). We exploit this to evaluate the probability that a cell will be in a certain genetic activation pattern, in the long run. By this, we can infer a measure of the average time needed to reach a stable GRN configuration, thus estimating the cell cycle length (Figure 4, left).

In formulas, if 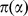 is the stationary probability of a pattern 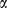, we define the length 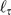 of the cell cycle for a cell of type 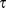 as
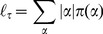
(5)where 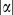 is the number of genetic configurations of the pattern 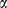 (i.e. number of states of the attractor), which ranges over the set of patterns (i.e. attractors) belonging to the considered ergodic set.

The length of the cell cycle is then an emergent property of the NRBN dynamics, thus a conversion between the involved time-scales is required; this is, to the best of our knowledge, a novel result. We link the internal time-scale (i.e., the NRBN steps) to the external one (i.e., the MonteCarlo steps) by considering that 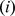 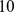 MonteCarlo steps correspond to 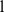 hour of biological time, according to [17], and that 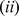 the *average* length of a cell cycle is in between 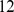 and 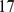 hours (we here arbitrarily choose 150 MonteCarlo steps, namely 15 hours, as a reasonable value to be used in the conversion) (ibidem). Thus, since the natural unit for 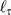 is the NRBN step, we have the following conversion:
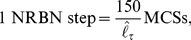
(6)where 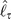 is the *average* cell cycle length of all the cell types of the NRBN. In this way, the relative difference in the lengths of the cell cycles accounts for the difference in the replication pace of the distinct cell types, as a consequence of the emergent dynamics of the GRN. So, for instance, if a cell has only two cell types of length, respectively, 2 and 10, the former type will require 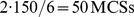 to complete the cell cycle, whereas the latter will require 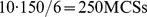.

#### Cell size dynamics

As we stated above, each cell of type 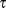 grows towards a target area 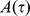, and newborn cells have assigned area 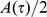 so they need to double their size before performing mitosis (Figure 4, top). To spontaneously drive a cell to double its size we make the target area to be time-dependent on the time-scale of the internal GRN, denoted 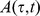. As if it was *mechanically isolated*, the time-dependent area grows linearly when the cycle starts at some time 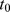, that is
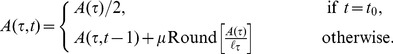
(7)


Here we discriminate among proliferative (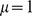) and non-dividing (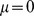) cells; with reference to the tree in Figure 1, non-dividing cells are paneth, goblet, enteroendocrine and enterocyte. Also, 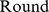 denotes the nearest-integer function. By introducing this time-dependent area we refine the constraint area term of Equation (3) to be 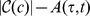, where 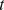 is time passed since the beginning of the cell cycle for cell 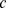.

#### Cell division and differentiation dynamics

As long as the CPM dynamics goes on, so does the underlying GRN dynamics within each cell, in terms of dynamical evolution of the gene activation patterns. We hypothesize the existence of a certain level of biological noise and random fluctuations, which induces a number of gene mutations: the mutation rate 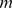 defines the frequency of single flips of genes (as when computing the ergodic sets) and is derived from experimental evidences [80]. In this way, cells that are characterized by TESs with more than one attractor may wander through the distinct gene activation patterns, by means of random mutations.

When a cell concludes in 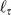 NRBN time-steps its cycle and reaches its target size 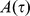 on the CPM, it instantaneously divides and differentiates (Figure 4, right).

As explained in the previous section, once cells differentiate they increase their noise resistance threshold [30]. The differentiation branch depends on the dynamics of the underlying GRN, as previously discussed and, in particular on the specific gene activation pattern in which the cell is located when the cell divides. Notice that stem cells perform *asymmetric cell division* to preserve their niche, i.e., only one daughter cell differentiates, the other one remains a stem cell [1].

### Comparison with Wong's differentiation model

The cell differentiation process modeled here profoundly differs from the one in [17]. First, we here consider a branching (lineage) tree in which the fate decisions of newborn cells depend on a random process (see Figure 3). Also, our random process is ruled by the level of biological noise and by the constraints emerging from the dynamical properties of the gene activation patterns such as their reachability and robustness against perturbations (see Figure 4). Conversely, in [17] there is no branching, i.e. a parent cell can generate only one type of descendent and, hence, the fate decisions are deterministic for every newborn cell.

Second, in our model Paneth descend from stem cells (via asymmetric differentiation), while in [17] Paneth cells are independent of the lineage tree. More in general, the two approaches consider different cell types.

Third, as a consequence of the multiscale link required by our model, we reduce the model parameters by letting emerge, from the internal dynamics, many properties of cells such as cycle length and growth rate, which are prefixed in [17] (see Figure 4). As a whole, less *a priori* assumptions are considered in our model, and thus our model is more general and flexible. In this respect, the differentiation process presented in [17] is a very particular (and constrained) case of the model hereby introduced. This has also repercussions on the interpretation of the results (see the Results section).

Fourth, we clarify that solely the explicit presence of a gene networks allows to investigate the role of perturbations on the overall dynamical behavior, thus making our model amenable at different analysis than those in [17], with particular regard to the issue of cancer development. Similarly, signaling pathways, as those driving cell adhesion properties (which are now implicitly included in the model through the cell adhesion matrix), may be explicitly inserted in the model by introducing, for instance, chemical gradients influencing the activity of certain genes of the GRN and linking the activity of those genes to the adhesion properties of the cells. This will eventually allow to study the influence of alterations hitting these pathways.

Finally, notice that our approach is general and might be applied to lower-level representations of GRN, especially if entities (genes, proteins, RNAs, etc.), connections (regulation and signaling pathways) and functions (interaction rules) of a specific organism were indeed available. In fact, the theory of Threshold Ergodic Sets could be used in different settings to determine the emergent lineage tree of, for instance, quantitative models. Clearly, the detection of the *relevant realistic* entities and interactions involved in crypts is a goal deserving its own research, and out of our scope in this work. Nonetheless, our multiscale approach sets the basis for a novel view on how the *dynamical* properties of GRNs may be related to the phenotypic properties of cells and tissues, possibly shedding a light on their complex interaction.

## Results

Simulations of the model were performed by a ad-hoc Java implementation developed by our research group. The search of the NRBN matching the tree in Figure 1 was performed by using GeStoDifferent, a Cytoscape [81] plugin to generate and to identify GRNs describing an arbitrary stochastic cell differentiation process [82].

Most of the parameters of the model are set on the basis of experimental data on *mice* and on the general biological knowledge concerning intestinal crypts, whereas the remaining ones are estimated to fit the overall dynamics, with regard to both the spatial and the GRN models. Tables 1 and 2 show the parameters used in the simulations.

We specify that some of the analyses that will be presented reproduce some of the results shown in [17], in order to compare the distinct approaches to the modeling of crypts.

### Properties of the suitable GRNs

As mentioned above, the number of NRBNs with emergent behavior coherent with the crypt lineage commitment tree must be low. Further, no constructive approach is known to determine such networks, and a generative approach is then required.

We here limited our search to NRBNs with certain *structural* features (summarized in Table 1) known to be plausible for real GRNs. In particular, we used *scale-free* topologies [83], i.e. NRBNS where the fraction of genes with 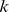 outgoing connections follows 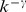 for large 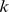. Here we used 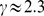 estimated to be a realistic value for many biological networks, including GRNs [84]. We designed networks with 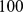 nodes, a number that is reasonably in line with the order magnitude of high-confidence cancer driver genes recently identified in various tumor types, among which colorectal cancer [85]. Even though in the current analysis we describe the normal functioning of crypt, this choice will allow to investigate the relation between alterations at the GRN level and the emergence of aberrant structures and phenomena, also permitting to include in the model portions of real architectures involving genes related to cancer development. Finally, concerning boolean functions, we used biologically plausible *canalizing functions* [86], [87].

Our results confirm that finding suitable NRBNs is indeed hard: only 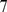 out of 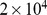 (i.e. 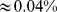) distinctly generated networks are amenable at use. This confirms that even rather small networks can display a broad range of dynamical behaviors, thus finding the correct emerging lineage commitment tree is hard. This outcome also points to a strong Darwinian selection process at the base of the emergence and evolution of the current human GRNs. We tried to statistically discriminate among these NRBNs by evaluating some measures commonly used in network analysis (see, e.g., [83]): the number of emerging activation patterns (i.e. the number of attractors), the average number of genetic configurations they contain (i.e. the length of the attractors), the clustering coefficient of the network, its diameter, the average path length and the average bias of the boolean functions. Nonetheless, even if the number of suitable NRBNs is too limited to draw definitive conclusions, the comparison hints at the lack of appreciable differences among the suitable and unsuitable networks (not shown here). Further, this suggests that identifying some GRN parameters to improve this generative approach is indeed hard, as expected by considering that real GRNs are the result of a Darwinian selection process which selected the fittest networks in terms of robustness, evolvability and adaptability to dynamic environmental conditions.

As explained in the previous sections, the emergent properties of the GRN are related to some key features of the cell cycle and differentiation processes at the spatial level. In particular, in Table 3 we show the cell cycle lengths, as computed with equation (5) for the 7 suitable GRNs actually used in the simulations.

**Table 3:** Cell cycle length 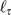 and its average value 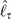, in NRBN steps, as computed with equation (5) for the 7 suitable GRNs used in the simulations, divided by cell type.

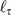	*Net_1_*	*Net_2_*	*Net_3_*	*Net_4_*	*Net_5_*	*Net_6_*	*Net_7_*	Average
St	5.47	6.50	6.17	1	20	7.23	4.35	7.24
TA1	6.60	7	5.62	1	20	9.8	4.81	7.73
TA2-A	8	7	4	1	20	7.50	6	7.64
TA2-B	6	7	14	1	20	13	2	9
Pa	4	1	2	1	20	4	2	4.86
Go	6	7	14	1	20	13	2	9
Ec	8	7	4	1	20	7	6	7.57
Ee	8	7	4	1	20	8	6	7.71
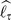	6.58	6.07	5.68	1	20	7.97	4.45	

It is possible to notice that the length of the cell cycle ranges from 1 to 20 NRBN time steps in different nets and that the variance can be dramatically different among nets, ranging from the case of networks in which all the cell types have the same cell cycle length (i.e. same replication pace), to the case of very different lengths (i.e. very different replication paces). By looking at the average values one can see that most of the cell types have a similar cell cycle length, around 7. Considering that in simulations we set 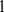 NRBN step 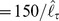 MCSs, we can estimate that on average 21 MCS, i.e. around 2 hours, are needed in order to switch among the configurations of a gene activation patterns (i.e. from one state to the following in the attractor). Accordingly, the average cell cycle lasts around 15 hours, which is set to be in accordance with biological knowledge (see the Biological background section). Surprisingly, cell types that are closer in the tree (i.e. 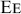 and 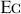) display almost identical cell cycle length with every network, pointing at an interesting property of such a system.

Distinct other properties of the gene activation patterns of the suitable networks are reported in File S1. We here remark that a rather large variability in the robustness to perturbations of the patterns is observed in the different cases, ranging from patterns that are almost imperturbable (99% of the single-flip perturbations end up in the same pattern) to ones that allow switches to other attractors in 30% of the cases after single flip perturbations. This result hints at interesting research perspectives related to the possible advantage for GRN of being *sufficiently* robust to perturbation, while not being too ordered. Historically, it has been hypothesized that natural evolution might favor biological systems that operate in the so-called *critical* dynamical regime, i.e. the phase state between the ordered and disordered behaviors, as defined in complex systems research [31], and this because of the optimal trade-off between robustness and evolvability. In particular it was suggested that gene networks may operate in, or close to, such a critical state, also according to some experimental evidences, provided for instance in [31], [34], [36]. In our case, the analysis of the stationary distributions shows very different scenarios, ranging from the case in which all the patterns are almost equally probable, to that of networks in which some of the patterns are very unlikely (e.g. less than 5%). Also in this case, it would be interesting to match these results against experimental evidences, to investigate the role of the temporal permanence within the same pattern and of the transitions among them.

### Cell sorting and overall homeostasis

The major goal of this work is to determine under which conditions the correct functioning of intestinal crypts is ensured and maintained, with particular reference to *cell sorting*, *coordinate migration* and *general homeostasis*.

To this end, we analyzed the crypt dynamics via CPM simulation, by using the suitable NRBNs. Please refer to Tables 1 and 2 for the parameters of the CPM used in the simulations. To account for the role of the initial displacement of cells within the crypt we tested 4 distinct configurations on a 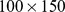 pixels lattice, according to the initial level of “order” (in order to represent the spatial properties of the cells with an adequate resolution, we set 1 pixel side to 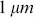, so to have crypts of size 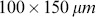, which is in agreement with experimental evidences [6], [19]). A *disorder* parameter, 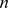 discriminates the first three configurations: 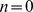 denotes a configuration in which the cells are perfectly sorted, 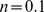 (resp. 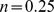) a configuration in which 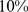 (resp. 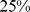) of the cells are randomly positioned on the lattice. The fourth initial condition is composed only of stem cells, positioned at the bottom of the crypt, while the remaining lattice is empty. The latter configuration aims at investigating *in-silico* the dynamics of isolated stem cell progeny populations, as classically done via *in-vitro* experiments [88].

**Figure 5:**
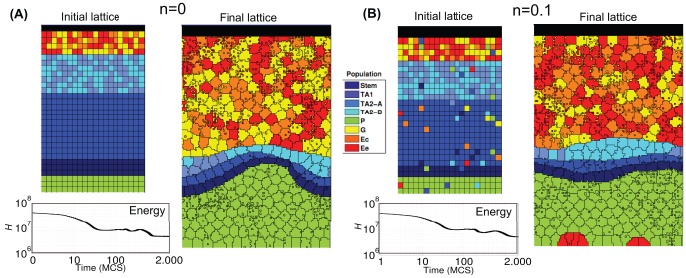
Crypt homeostasis - 1. Initial lattice configurations for 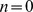 (**A**) and 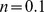 (**B**) and corresponding lattice after simulating 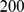 hours of crypt evolution, for a single simulation. The overall system energy is the average of 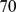 independent simulations. Crypt layout was drawn by using the visualization capabilities of CompuCell3D [107].

**Figure 6:**
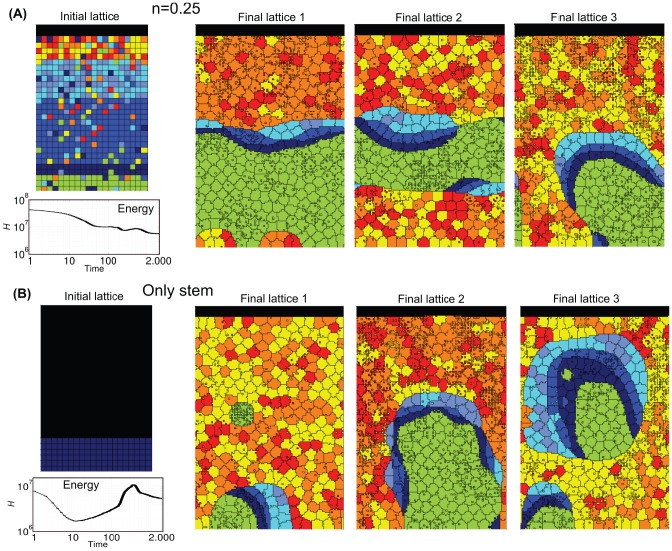
Crypt homeostasis - 2. Initial lattice configurations for 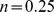 (**A**) and the case of only stem cells (**B**), and corresponding lattice after simulating 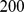 hours of crypt evolution, for a single simulation. The overall system energy is the average of 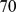 independent simulations.

In all the initial conditions cells are assigned a square shape, in the first three cases 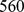 cells are displayed with the following cellular proportions: 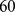 stem cells, 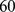 Paneth, 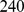 TA-1, 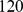 TA-2 and 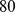 differentiated cells. In the fourth case 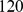 stem cells are considered. Clearly, the initial squared shape of the cells is a strong simplification, which however does not affect our analysis, because the energy minimization-driven dynamics leads the cells to more physically plausible shapes in a few MCSs. The initial conditions are shown in Figures 5 and 6, together with some sampled crypts after 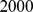 MCS (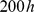) with 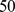 final *annealing steps*. It is known that, by performing simulations at nonzero temperature, cells are not required to be connected and cell boundaries can crumple, especially when the temperature is comparable to the boundary energy. Glazier and Graner suggest to use a certain number of zero-temperature annealing steps to remove these defects, even if this procedure evolves the spatial pattern as well [26]. Nonetheless, we here remark that this kind of lattice artifacts are not relevant to our analysis, which is based on the statistical analysis of quantitive measures at a coarser grain. For each of the 7 suitable GRNs we performed 10 independent CPM simulation runs, in order to have a relevant statistics. We remark that the values of 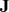 are based on experimental results showing that a high activation level of the Eph receptor reduces cell adhesion and vice versa [65], (see Table 2). Only the relative magnitudes of cell adhesion energies are needed to our modeling approach.

By these figures it becomes clear that the final crypt ordering is dependent of the initial ordering. In particular, for very low-noise configurations the correct crypt behavior always emerges. Differently, in the case for 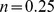 deeply different scenarios are displayed at each simulation. In some cases, the correct cell stratification is achieved, while in others some distinct geometrical shapes, e.g., encapsulations and invaginations, are observed, and the overall homeostasis is not achieved. In the fourth initial configuration (i.e. only stems), it seems unlikely that the crypt may reach a correct stratification. In the next sections we analyze these scenarios in detail by evaluating specific statistics.

Notice that the overall system energy (i.e. the Hamiltonian 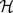), whose variation in time is shown in the figure, asymptotically reaches a minimum value which ensures an optimal (dynamical) configuration of the cells on the lattice. In the specific case of stem cells (Figure 6), one can observe a peak in the Hamiltionian after around 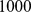 MCS. This phenomenon is due to the expected progressive appearance of large populations of distinct differentiated types, as opposite to the relatively more favored initial configuration, in which only cells of a unique type (i.e. stem) are present in the system.

One of the most important results of these (and the following) analyses is to show that in our model the stochastic differentiation at the GRN level is itself sufficient to ensure the *normal* activity of the crypt, in terms of overall spatial dynamics. This result is even more surprising by considering that, as shown in the previous section, the lengths of the cell cycles are indeed different in the distinct suitable networks used in the simulations. Hence, it is reasonable to hypothesize the existence of a relatively broad region of the gene activation space in which the correct functioning of the crypt is maintained, despite the differences in the replication pace of different cell types, as long as a suitable differentiation tree is maintained to ensure the correct cell turnover. Besides, with this approach no explicit signaling pathways are considered, which instead result from the interplay between the GRN and the CPM features. Interesting research perspectives derive from this outcome, with particular regard to the configuration of the activation patterns related to the emergence of *aberrant* structures.

### Cell population dynamics

**Figure 7:**
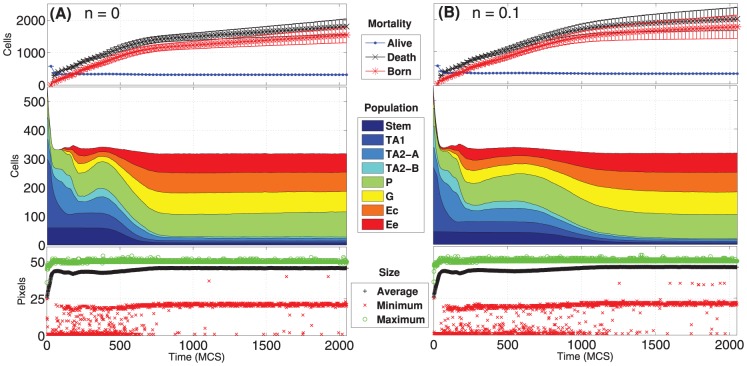
Dynamics of the cellular populations - 1. Number of cells for each cellular population (cumulative), number of newborn, dead and alive cells and maximum, minimum and average cell size, in time. Notice the prediction of 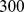 cells, regardless of the two initial conditions 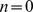 (**A**) and 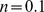 (**B**). The length of the transient is similar, in both cases.

**Figure 8:**
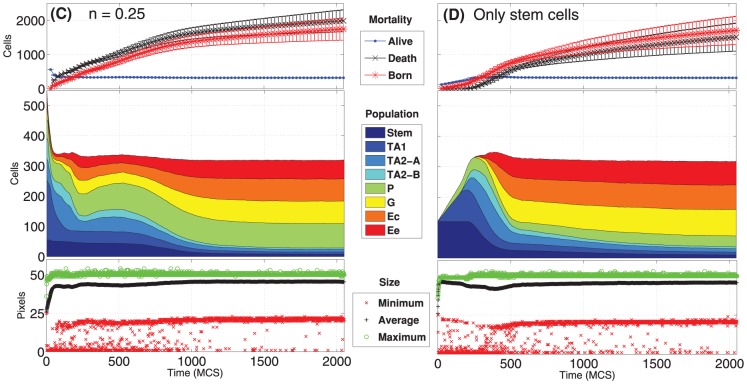
Dynamics of the cellular populations - 2. Number of cells for each cellular population (cumulative), number of newborn, dead and alive cells and maximum, minimum and average cell size, in time. Notice the prediction of 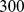 cells, regardless of the two initial conditions 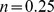 (**C**) and the case with only stem cells (**D**), with a longer transient.

The variation in time of the *number of cells in each population* is shown in Figures 7 and 8 for the four distinct initial configurations. Despite some differences, in all the cases an asymptotic stable proportion is reached, after a transient in which the crypts tend to adjust. In particular, a proportion between the cell types is maintained in all the cases, predicting quantities that are in agreement with what is supposed to be the general proportion of cell populations in real crypts, i.e. around 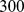 [1], [17], [19], [89]. More in detail, the *average* final configuration involves cell population in these proportions: Stem cells 2.5%, TA1 2.5%, TA2-A 2%, TA2-B 2%, Paneth 27%, Goblet 22%, Enterocite 22% and Enteroendocrine 20%. Surprisingly, this pseudo-equilibrium is reached regardless of the different initial conditions, suggesting that the GRN-driven crypt dynamics is able to ensure a “correct” cellular proportion. The only clear difference predicted by the initial conditions is that, in the case of a crypt with only stem cells, the system appears to have a longer transient.

In the same figures we also show the *number of newborn* and *dead cells* (either due to apoptosis or to the expulsion in the intestinal lumen). Even these two quantities tend to a dynamical equilibrium for all the distinct initial conditions, hinting at an intrinsic capability of the system to ensure a *correct dynamical turnover* or, in other words, the *renewal of the tissue*. The quantities shown in the figures agree with the phenomena supposed to characterize real crypts (see [17] and references therein).

Finally, the maximum, minimum and average *size of each cell* are shown. We remark that the initial cells are newborn, so their size is half of their target area when mature, i.e. just before undergoing mitosis. This is the reason why all the initial values of this statistics, particularly in the average case, are much lower than the asymptotic ones which are, in any case, stable. One can see that the average cell size is very close to the maximum, suggesting that the crypt mostly contains “adult” cells. Also, being the variance relatively small, this suggests that cells have similar sizes, on the average. Finally, the maximum size has an upper bound proportional to the pre-mitotic size, which is only rarely exceeded due to random fluctuations.

We here remark that these results imply stronger conclusions than those shown in [17], especially considering our more general differentiation model. In fact, the size of the cell populations and their proportion is not granted by the differentiation tree, and are thus emerging as a result of a fate decision stochastic process. In particular, the newborn cell type probability, the length of the cell cycle and the rate of growth and duplication, which emerge from the properties of the underlying GRN, can be very different among cell types (e.g., see Figure 4). Therefore, it is noteworthy that the cell population proportion is stable and “correct”, together with other key homeostasis measures, for different TESs landscapes. This result could suggest that the homeostasis of this system, as modeled and measured with the current analyses, is relatively insensitive to the variation of certain key cellular properties related to the stochastic differentiation process.

#### Stem cells population dynamics

We briefly comment on the population dynamics of stem cells. As above recalled, it is currently hypothesized that mammalian intestinal stem cells are firmly located at the base of the crypt, as suggested by analyzing the *Lgr5* expression marker [90]. However, in a very recent study Ritsma *et al.* tracked *in-vivo* the short-term spatial dynamics of intestinal stem cells by using continuous intravital imaging of *Lgr5-Confetti* mice, reporting of stem cells being expelled from the niche [91]. In particular, it was discovered that certain stem cells are sometimes passively displaced from the upper boundary of the niche in the region of transit amplifying cells, while not loosing stemness and not entering the transit amplifying stage (Figure 6 in [91]). The authors also suggested that this peculiar spatial dynamics may be due to the competition for space derived from the cell proliferation dynamics, which would lead some stem cells to be displaced from the niche independently of their division history. In other words, some stem cells may be characterized by a survival advantage only due to their specific position in the niche, while other may have a bias towards loss.

This “expulsion” phenomenon is observable in our model. In fact, despite the (constrained) asymmetrical division (i.e. the *invariant asymmetry* division mode), the number of stem cells actually decreases in time (Figure 7 and 8), hinting at an expulsion process and at a successive migration of some stem cells in the proliferative and differentiated regions, previous to their dispersion into the lumen. Notice that the experiments in [91], which focus on the short-term dynamics of this phenomenon, do not show the final shed of the stem cells in the lumen. It can be hence hypothesized that in our model the competition for space and resources, which result from the interplay between the energetic constraints at the spatial level and the GRN-driven cell cycle and proliferation dynamics, drives stochastically the system towards a configuration in which some stem cells are maintained in the niche, while others are expelled. In all the analyzed configurations a progressive reduction in the number of stem cells is observed, with a certain variance. We remark that this phenomenon is emergent in our model.

From another perspective, the asymmetrical division of stem cells, as modeled here, is insufficient to ensure the maintenance of the stem cell niche and population, which is profoundly affected by this multi-level interplay. In particular, the fact that all the tested configurations tend towards a stable and similar plateau might suggest that the system is able to self-organize towards an “optimal” proportion of cell populations and, accordingly, of stem cells. All these considerations affect also the clonal dynamics of crypts, as we discuss below.

### Coordinate migration

**Figure 9:**
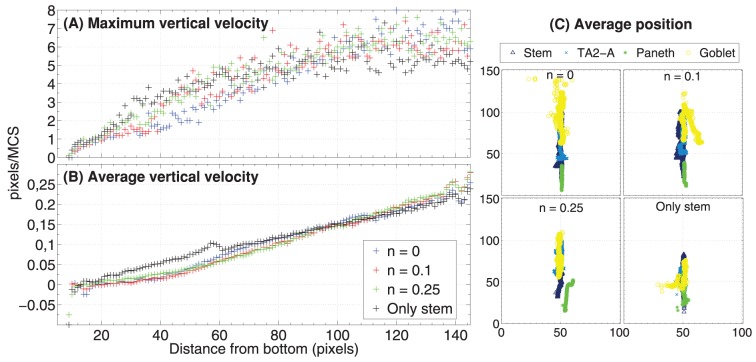
Cell migration. Relation between the distance from the bottom of the crypt (in pixels, i.e. 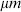) and the maximum (**A**) and average (**B**) vertical velocity of the center of mass of all cells. This is averaged for all simulations, at all the time steps. In (**C**) we show the average center of mass for stem, Goblet, Paneth, TA2-A, averaged on all simulations and sampled every 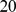 MCS.

From experimental results it is known that cells at the bottom of the crypt move slower toward the top than cells positioned in the upper portion [17], [92]. By looking at Figure 9 we can notice that there is a correlation between the distance from the bottom of the crypt and the average *vertical velocity* of cells. Only the *vertical component* of the velocity is shown there, the positive values being associated to the direction toward the top of the crypt. We do not show the minimum vertical velocity which is 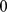, for some cells and we also remark that the average values do not take into account the fact that some cell populations (e.g. stem cells) move much less than others (e.g. differentiated cell), as required. Recalling that 1 pixel side, 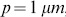, the average vertical velocity of cells ranges from 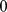 pixel/MCS at the bottom of the crypt to 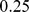 pixel/MCS at the top, that is at most 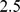 pixel/hour. Hence, we can estimate the average time needed for a random descendent of a stem cell (and originating in the stem cell niche), to complete the (progressively faster) migration toward the lumen. It turns out that *around* 650 MCS, i.e. 65 hours, around 3 days, are needed and this result is in perfect agreement with experimental data [1], [93]. We can also notice that the maximum observed vertical velocity ranges from around 0 at the bottom of the crypt to around 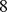 pixel/MCS at its top, with regard to all the configurations. This outcome indicates that some cells can move dramatically faster than other in the overall spacial displacement, due to local energy configurations.

In order to highlight the relative positioning of cell populations during a simulation, in Figure 9 one can see the movement of the average center of mass of the cells belonging to four distinct types, i.e. stem, Paneth, Goblet and TA2-B, during the whole simulation. A general correct positioning of the populations is maintained with all the distinct initial configuration, yet as long as the level of disorder increases the displacement becomes less precise, as for the case of only stem cells. Notice also that the average position of the stem cell population, approximately at the bottom of the crypt, suggests that the observed process of expulsion of certain stem cells from the niche (see above) is much faster than their residence time. In general, a coordinate migration involving the whole crypt is proven to be an emergent property of the GRN-driven dynamics.

This and the subsequent analyses prove that cells translocate in a coordinate fashion towards the top of the crypt, as observed *in-vivo* [93].

### Quantitative measures of spatial ordering

Experimental evidences suggest that epithelial cells migrate in coordination as sheets in culture [94]. Along the lines of [17] we determine whether our cells move coordinately by using the following *spatial correlation index* [94]:
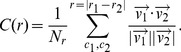
(8)


Here 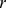 is the distance between the center of masses of two generic cells 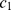 and 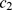, 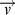 is their cell velocity and 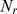 is the overall number of cell pairs with distance equal to 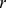. If a inverse reciprocity relation holds between 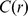 and 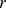 this implies that closer cells display a more coordinate movement than distant ones.

**Figure 10:**
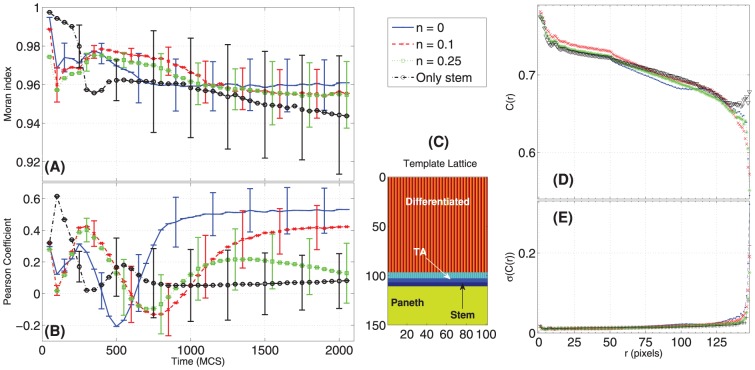
Spatial statistics for crypt stratification and coordinate migration. Time-variation of the Moran Index, MI (eq. 10) (**A**) and of the Pearson Coefficient, PC (eq. 11) (**B**). In (**D**) the Spatial Correlation 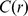 (eq. 8) with the relative standard deviation (**E**) is displayed, for the four initial conditions. A crypt can be considered well stratified if its MI is high (it is indeed stratified), and its PC is high (it has the cellular populations in the correct order), according to a template (**C**).

This is what we actually observe in Figure 10: the movement of the cells is highly correlated, unless for very distant cells, which also show large fluctuations. For the stem cells case we observe a slight decrease in the average correlation. This outcome closely resembles the one shown in [17], confirming that the coordinated cellular movement is maintained when also a GRN is used to drive the stochastic differentiation dynamics.

In order to automatize the evaluation of the general *spatial order* of crypts, we propose to use the *Moran Index* (MI, [95]) and the *Pearson's correlation coefficient* (PC) [96]. These measures will allow to understand if the cell populations form groups, and if the correct stratification is achieved. We recall their definition here, extended straightforwardly to matrices (originally, these measures are defined for vectors); the PC 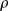 for two matrices 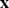 and 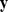 is a function of their (co)variances
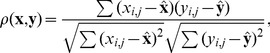
(9)where 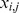 is a component of 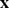, and 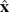 is its average. The PC ranges from 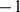 (inversely correlated) to 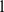 (correlated) and at 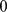 there is no correlation between 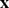 and 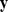.

The PC is also used in the MI, which is used to determine if lattice positions are correlated, that is if cells are likely to form strains of the same type. To define the MI we associate, to each cellular type 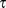, a unique integer value (so 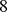 values in total), and we evaluate, for each position, the average of all its neighbor cellular types. In formulas, for a position 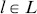 we evaluate
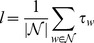
where 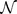 is the set of neighbors of 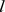 in 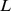 (we used the 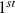 order Von Neumann neighborhood, i.e. 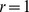), and 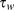 is the integer associated to the cell type in 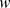. This formula yields a new lattice 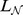 to compute the MI as
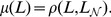
(10)


Notice that 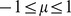 with the usual meaning, and that the MI is equivalent for two symmetrical lattices. Thus, despite being a good measure for aggregation, the MI itself does not distinguish if a crypt is stratified with the correct bottom-up ordering, or, for instance, if it reversed. We can anyway use a *template lattice* 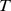, i.e. a lattice were the cellular stratification is made explicit, to asses, the PC between a lattice and the template, that is
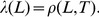
(11)


By combining these measures we can state that a crypt is well stratified if it has high 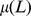 (i.e. it has high MI thus it is stratified), and it has high 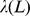 (i.e. it is highly correlated to the template, thus it has the cellular populations correctly stratified).

All these spatial measures are plot in Figure 10. Initially, the MI (averaged over all the simulations) is clearly dependent on the lattice initial condition. After a transient where the stratification level decreases, the MI asymptotically approaches a high value (still proportionally to the initial level of noise), in all but the only-stem-cells case, where the MI gets highly dispersed. This suggests that the stratification is generally maintained, with distinct GRNs and initial conditions, in all cases but when only stem cells are present, a clearly particular scenario.

We compared these statistics with the PC for the corresponding simulations and the template lattice shown in Figure 10. The template lattice considers the proportion among cell populations that is derived from the average final configuration of the correctly stratified lattices (see above). The variation of the PC in time seems to be dependent on the initial condition, thus giving further information besides the “general” degree of order depicted by the MI. The PC suggests an inverse proportionality between the initial noise and its asymptotic value, thus hinting at the importance of the initial crypt morphology for its development in the preliminary stages. As for the MI, the lowest PC is for the lattice with only stem cells since we do not impose any constraint on the spatial development of the crypt besides upper/lower bounds. Movement direction and expansion of the cell population emerges from the dynamics induced by the underlying GRN.

### Clonal expansion

To investigate the process of *clonal expansion* in crypts we can track the descendant of each stem cell. This will help to determine, in future works, whether any relevant difference is detectable with respect to the case of cancer evolution. It is in fact known that tumors develop through a series of clonal expansions, in which the most favorable clonal population survives and begins to dominate, in a “survival of the fittest” Darwinian selection scenario [97].

As far as the normal tissue development is concerned, it was proposed that stem cells may be routinely lost and replaced through a stochastic process [98]. Among various hypotheses, it was suggested that such a process may be driven by noisy gene expression, leading to cell-to-cell variability in response to environmental changes [99]. Besides, distinct experimental evidences suggested that also developing tissues are ruled by transcriptional noise to generate stochastic fate outcomes [100]. Our model accounts for this specific phenomenon by relating noise-resistance, stochastic gene activation patterns and, accordingly, the cell cycle and the cell fate decision processes.

In our model we consider *asymmetric stem cell division* (i.e. the *invariant asymmetry* mode) as observed, e.g., in hematopoietic cells [101], [102]. However, distinct differentiation modes could be included in the model as well: for instance, the *population asymmetry* hypothesis (either cell-autonomous or external-induced) states that stem cells descendants might either differentiate or remain in a stem state, in a process coordinated by the requirements of the tissue [98], [103]. Population asymmetry is then characterized by *neutral competition* among the clones.

The results presented in this section allow to investigate the clonal expansion phenomenon in the development of healthy crypts when invariant asymmetry is considered. Every stem cell and its descendants constitute a *clone*, which is *alive* if at least one of its constituting cells is alive. Notice that whenever a large number of proliferative and differentiated cells is present in the initial configuration of the system (see Figure 5 and 6) we consider solely the clones constituted from the stem cells present in the initial configuration and their future descendants. This because it is not possible to associate non-stem cells to such clones in a non-arbitrary way.

#### Stem cell and clone survival probabilities

As discussed above, some stem cells and their clones are characterized by a survival advantage, as shown in Figure 2 of [91] where the survival probability of clones decreases in time, whereas the average clone size increases. These results are differently reproduced in our model according to its initial configuration.

**Figure 11:**
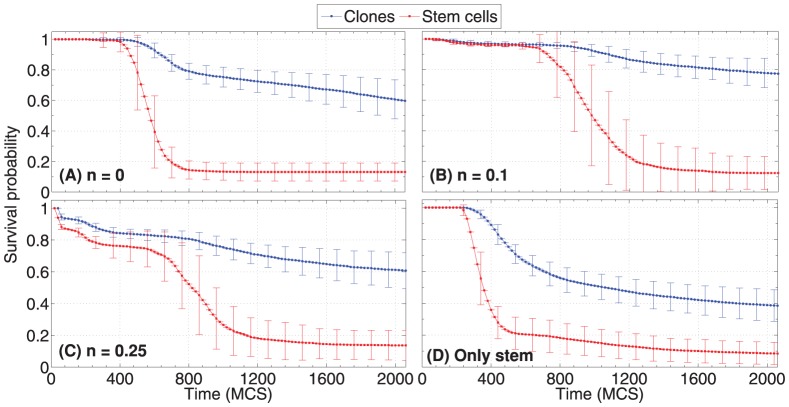
Survival probability of stem cells and clones. For each of the initial conditions in Figure 5 and 6 and for all the simulations we evaluate the average and the standard deviation of the survival probability of stem cells and clones, as the ratio, over time, of alive stem cells and clones over the initial number of stem cells.

In Figure 11, in all the configurations, the variation in the survival probability of stem cells and clones tend to a similar plateau with around 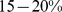 of the initial stem cells. This is due to the aforementioned phenomenon of progressive expulsion of some stem cells from the niche, in a scenario of competition for limited spatial resources. In general, this hints to the existence of an optimal number of stem cells ensuring the correct functioning of the system, regardless of the initial configurations, which are characterized by different transients. In a future study we plan to correlate, in an automated hypothesis-testing scenario, the predicted plateau value (i.e. number of stem-cells) to statistical measures of crypt homeostasis, possibly estimating optimal “proportions” of stem cells.

The survival probability of clones shows an analogous overall behaviour, yet with much higher magnitudes, displaying average final configurations (at 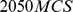, around 200 hours) with a relatively high fraction of alive clones, around 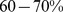 for the normal configurations and 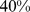 for the only stem case. This latter result suggest that in the only stem case a lower number of clones colonizes the crypt (see the conclusions below). A possible explanation of the difference between the survival probability of stem cells and the relative clones can be given by analyzing the clone size dynamics.

#### Clone size dynamics

We estimate an upper bound to the number of cells belonging to a clone generated by a stem cell. Let us assume a hypothetical scenario with 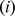 no spatial competition and unlimited available space, 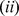 a non-skewed differentiation tree with depth 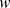 and 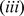 synchronized pace of division for all the involved cells, the *clone size* 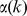 after 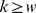 cell cycles grows approximately as:
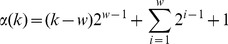
(12)where the first term accounts for fully differentiated cells and the second for the others. In our model spatial competition and finite space play a key role in the overall dynamics (see above), the tree in Figure 1 is skewed because of the Paneth cells branch and the pace of division is not synchronized, so 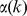 is an approximate upper bound for the clone size growth. Yet, it explains why relatively large clones can be observed in a limited number of division rounds. Therefore, the probability that *all* the cells belonging to a clone are lost decreases as the size of a clone increases, e.g. some cells of the largest clones can progress and divide without being expelled into the lumen, and before entering apoptosis. Conversely, certain stem cells can be sometimes displaced from their niche, being progressively lost. Hence, the fact that a fraction of clones survive their own stem cell explains the differences in the trends of Figure 11.

**Figure 12:**
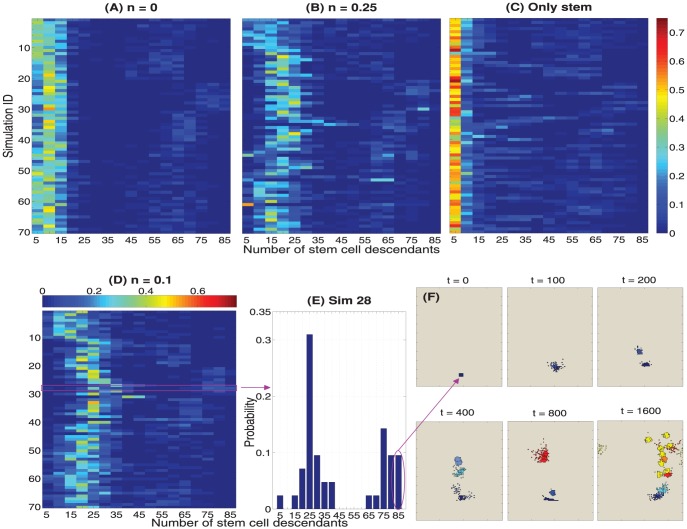
Stem cells descendants. Empirical discrete probability distributions of the number of *stem cell descendants* (alive and dead) assessed at the final simulation time: 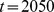 MCS. These heatmaps consider all the simulations and all the initial conditions: (**A**) 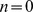, (**B**) 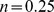, (**C**) only stems and (**D**) 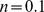. (**E**) is the histogram for the 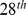 simulation with 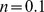. (**F**) shows the *alive progeny* of a stem cells with 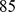 descendants at the instants 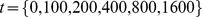, colored according to the cell types.

**Figure 13:**
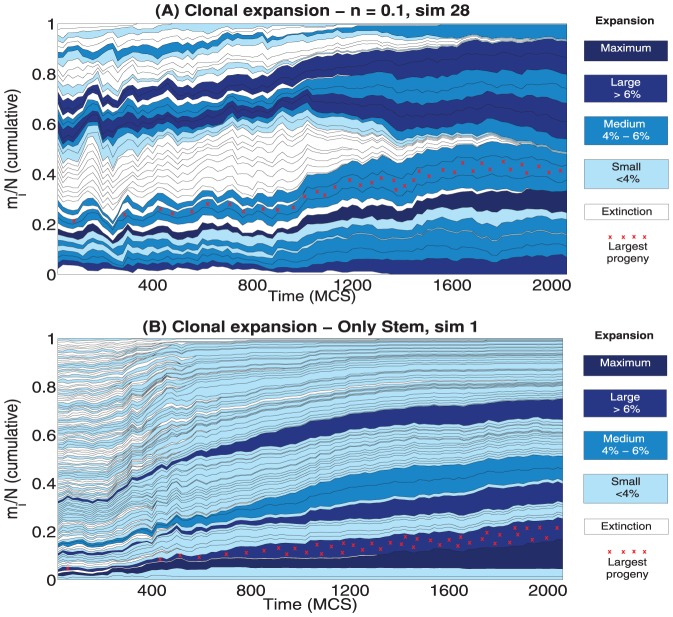
Clonal expansion, detail of two simulations. Variation of the cumulative clone density 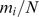 in time for the cases: 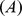 simulation 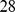 with 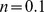, 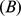 simulation 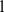 with only stem cells. The relative proportion of cells belonging to each clone over the total number of cells in the crypt at each time is shown. The blue gradient is related to different classes, accounting for the ratio of alive cells belonging to each clone at the end of the simulation. The red crosses mark the clone with the largest number of members along a simulation.

In Figure 12 we show the distribution of the number of descendants of each stem cell at the final simulation time, for each simulation. This quantity represents the potential *maximum size* of a clone in the case in which none of its cells are lost. Regardless of the initial configuration, a few cells only display around 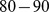 descendants, which is an approximate upper limit due to the maximum possible division rounds in the selected simulation time. Besides, because of the overall spatial dynamics, the total number of descendants dramatically differs from the number of alive clone cells. For example, one of the largest progenies is observed in simulation 28, case 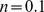. Despite the progeny includes 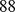 descendants, the clone actually fails in colonizing the whole crypt or even a significant proportion of it. In fact, at the end of the simulation only around 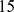 cells out of the 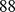 total are alive, that is only the 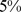 of the overall population (almost 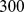 cells, see Figure 13). This phenomenon can be similarly observed in all the simulations. However, we remark that a larger number of descendants (on average) is predicted proportionally to the noise in the initial configuration, thus hinting to the importance of a correct stratification in maintaining the *right* pace of division in the crypt.

**Figure 14:**
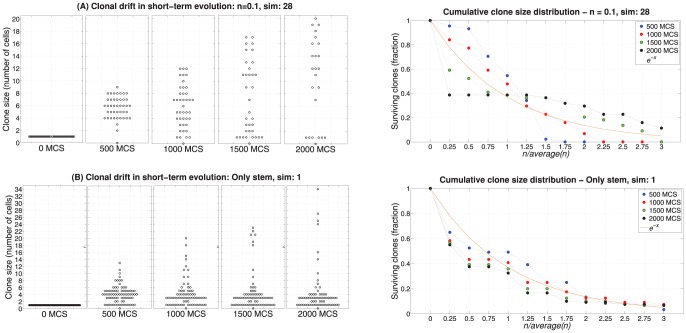
Clonal drift and scaling of the clone size distribution. On the left panels, we estimate at the instants 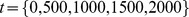 the number of cells constituting each alive clone for: sim 28, 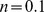, in Panels **A** and sim 1, Only stem case, in Panels **B**. On the right panels, we plot the proportion of alive clones for which the ratio between their size and the average clone size: 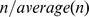 is larger than 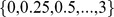, at the instants 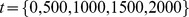. Also we show how the distributions scale with respect to 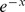.

We focused on two specific cases to highlight some interesting and commonly observed behaviors. In Figure 13 we show the (cumulative) variation 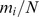 of the clone density computed over time where 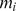 is the number of alive cells in the 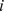-th clone and 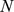 is the number of alive cells in the crypt at time 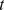, for two initial conditions. In Figure 14, as proposed in [98], we show for different simulation instants the size of each alive clone (i.e. the *clone drift*) and the cumulative clone size distribution, computed as the proportion of alive clones for which the ratio between their size and the average clone size is larger than a certain value. Panels A in Figure 13 and 14 refer to the 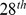 simulation with 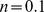, whereas Panels B refer to the 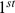 simulation with only stem cells in the initial configuration.

In the former case we identify a relatively large number of distinct clones (around 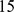) which share similar portions of the total population, i.e. from 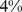 to 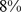, and in an apparently stable trend. Many clones actually get extinct during the simulation and a few others remain small. Also, the most prolific clone (shown in Figure 12 and 13) comprises of a low portion of the population, so no clone appears to be dominating. The outcomes in Figure 14 suggest a progressive enlargement of the clone size distribution resembling the experimental results on murine crypts shown in [98]. This suggests that also the invariant asymmetry scenario, as modeled with this multiscale approach, can actually describe the clonal dynamics in intestinal crypt, providing an alternative explanation to the population asymmetry hypothesis.

Nevertheless, this conclusion seems to be strongly dependent on the initial crypt configuration. In fact, in Figure 13 panel B, a few clones seem to start the colonization process of a crypt initially constituted of only stem cells. In this case, a specific clone at the end of the simulation already comprises of around the 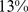 of the total cells, and appears to continue growing. Also, in this case the number of alive clones is much higher, as if they were in a sort of dormant phase, with the possibility of rising due to the stochasticity of the process. In this setting the system seems to be far from equilibrium and thus the possibility of a colonization by one or more clones is plausible. This results are mirrored by the clonal drift in Figure 14 and, mostly, by the cumulative probability of a clone to be larger than the average clone size, which is well fit by an exponential curve. Note that this result is rather common for crypts starting with only stem cells, as indicated by the lower clone survival probability for such an initial configuration, which hints at a lower number of clones progressively colonizing the crypt.

On the basis of these results we draw the following conclusions. Firstly, when crypts reach and maintain homeostasis, as we evaluated in the previous sections, the clonal dynamics appears to be “balanced” and not affecting the overall behavior. This conclusion is made stronger by considering that the clonal dynamics appears to be in a (slow) transient phase: even if the number and the size of the clones slowly changes, the overall homeostasis is maintained due to the underlying multi-level interplay.

Conversely, non-natural configurations of the crypt, as for instance when only stem cells are present in the initial configuration, can lead to both 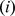 the emergence of aberrant structures, as seen in Figure 6 and 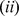 the appearance of dominating clones, in a complex interplay between the GRN and the spatial dynamics that still has to be deciphered.

In this regard, we expect that cumulative mutations hitting the underlying GRN of healthy crypts may lead to the appearance of fast replicant dominating clones, which may eventually colonize the whole crypt or a relevant part of it.

## Conclusions and Further Development

In this paper we introduced a novel multiscale model of intestinal crypt dynamics, by combining a well known in-lattice model from statistical physics to a boolean GRN model from complex systems theory. This model relies on a few assumptions only, thus reducing the number of its parameters, and the multiscale link between the crypt morphology and its genotype results from the emergent properties of the underlying GRN.

The model allows to efficiently investigate many dynamical properties of crypts such as, e.g., cell sorting, coordinate migration, stem cell niche correct positioning and clonal expansion. On the overall, the model suggests that the fundamental process of stochastic differentiation may be sufficient to drive the overall crypt to homeostasis, under certain crypt configurations. Our approach allows also to make precise quantitative inferences that, when possible, were matched to the current biological knowledge.

The model itself was conceived to be flexible and modular, thus all of its components will be possibly refined in future works, along the lines of other approaches (see the references provided in the introduction). In this first paper we focused on studying the development of healthy crypts, and we tried to assess the model conditions under which the activity of a normal crypt emerges and is maintained. These results will be used as a base for future research directions, all of them pointing to multiscale studies concerning the emergence of colorectal cancer, which is supposed to originate in crypts, most likely in the stem cell niche [2].

To this end, the choice of the internal GRN model allows for many possible improvements and research perspectives. For instance, along the lines of the usual NRBN approach, the effect of genetic perturbations of various types (e.g. *gene mutations*) will be assessed with respect to the emergence and development of cancer. Possible communication mechanisms among the GRNs of neighbor cells may be introduced in the model as in, e.g., [104], as well as more accurate descriptions of gene activation and dynamics as in, e.g., [105]. Also, the role of the extrinsic noise in the system, e.g. random thermodynamic and kinetic fluctuations, might be quantitatively assessed as discussed, for instance, in [106]. Besides, the role of further types of stem cell division beyond invariant asymmetry will be investigated.

Finally, the networks that we found suitable to describe the lineage commitment tree for crypts will be matched against the currently known portions of the human GRN by employing, for instance, *graph isomorphism* techniques. Also, current knowledge will be used to set up constraints on networks generation, possibly allowing to infer new portions of the human GRN related to the genes involved in the activity of the crypts. To address this ambitious goal, the relevant genes and their interactions involved in the evolution of colorectal cancer could be explicitly considered in the generation of the GRNs to be used in our model.

## Supporting Information

File S1 (PDF)
