# Supplementary material for: Investigating the Relation between Stochastic Differentiation, Homeostasis and Clonal Expansion in Intestinal Crypts via Multiscale Modeling
Source: PLoS One. 2014 May 28;9(5):e97272. doi: 10.1371/journal.pone.0097272 (PMC4037186; doi:10.1371/journal.pone.0097272)
Supplement: File S1 — (PDF) [file pone.0097272.s001.pdf]

Graudenzi et al. (2014): *Investigating the relation between stochastic differentiation, homeostasis and clonal expansion in intestinal crypts via multiscale modeling*

Supporting information

Glossary

internal model

GRN	*Gene Regulatory Network*	RBN	*Random Boolean Network*
NRBN	*Noisy Random Boolean Network*	TES	*Threshold Ergodic Set*
ATN	*Attractors Transition Network*	DTMC	*Discrete-Time Markov Chain*

morphological model

CPM	*Cellular Potts Model*	DAH	*Differential Adhesion Hypothesis*
ECM	*Extra Cellular Matrix*	MCS	*MonteCarlo Step*

cell types

St	*Stem cell*	TA1	*Transit amplifying stage 1*
TA2-A	*Transit amplifying stage 2A*	TA2-B	*Transit amplifying stage 2B*
Pa	*Paneth cell*	Go	*Goblet cell*
Ee	*Enteroendocrine cell*	Ec	*Enterocyte cell*

statistics

PC	*Pearson's correlation coefficient*	MI	*Moran Index*

Mathematical specification of the model

We here present the full mathematical specification of our multiscale model. With respect to the main text some notation might differ, when appropriate.

The lattice-based model of cellular tissue

Within a $n \times m$ 2D *lattice of positions* $L \in \{1, \dots, k\}^{n \times m}$ a population of k cells of any type is placed. We denote with $l_{i,j}$ the single-site variable (spin in CPM terminology) associated with position (i, j), we write $l_{i,j} = (c, \tau)$ if the position is occupied by a cell $c \in \mathcal{C}$ of type $\tau \in \mathcal{T}$, i.e. $\mathcal{T} = \{\text{St}, \text{TA1}, \text{TA2-A}, \text{TA2-B}, \text{Pa}, \text{Go}, \text{Ec}, \text{Ee}\} \cup \{\text{Ecm}\}$ being the finite set of cell types we consider plus a special type for the ECM. A cell $\mathcal{C}(c, \tau)$ with *current area* $a(c, \tau)$ is defined by

$$\mathcal{C}(c, \cdot) = \{l_{i,j} = (c, \cdot) \mid (i, j) \in L\}, \qquad a(c) = \sum_{l_{i,j}=(c,\cdot)} a_0 = |\mathcal{C}(c, \cdot)| a_0 \tag{13}$$

where a_0 is a basic area (in unit of pixels p) assigned to a single lattice position (a pixel).

The mutual interaction energy is defined via the Hamiltonian $\mathcal{H} : \mathbb{I}^{n \times m} \to \mathbb{R}$

$$\mathcal{H}(L) = h_{\text{DAH}}(L) + h_{\text{area}}(L). \tag{14}$$

Let $\mathcal{N}(i, j)$ denote the Von Neumann neighborhood of (i, j), and let $\tau_{i,j}$ denote the type of cell in $l_{i,j}$, the contribution to the energy according to the DAH is

$$h_{\text{DAH}}(L) = \frac{1}{2} \sum_{l_{i,j} \in L} \cdot \sum_{l_{x,y} \in \mathcal{N}(i,j)} \mathbf{J}(\tau_{i,j}, \tau_{x,y}) \Big[1 - \delta(l_{i,j}, l_{x,y})\Big]$$

where δ is the Kronecker delta function (which ensures that only the surface sites between different cells contribute to the adhesion energy), and $\mathbf{J}$ is the surface energy discussed in the main text. Let τ_c denote the type of cell c, the contribution to the energy of the area constraint is

$$h_{\text{area}}(L) = \lambda \sum_{c \in \mathcal{C}} \Big[a(c) - A(\tau_c, t_c)\Big]^2 \cdot \text{Heav}\Big(A(\tau_c, \cdot)\Big),$$

where the ECM has unconstrained area, i.e. $A(\textsc{Ecm}, \cdot)$ is constant and negative, and its constraint is suppressed by the Heaviside function Heav$(\cdot)$. Notice that here we made explicit the time-dependency of the area constraint by using t_c, the relative time since the beginning of the cell cycle for c.

Given a lattice L, we define the flip of a position (x, y) to a cell c to be the new lattice $L[c \leftarrow (x, y)]$ where

$$l_{i,j} = \begin{cases} (c, \tau_c) & \text{if } (i, j) = (x, y), \\ l_{i,j} & \text{otherwise}. \end{cases}$$

The CPM simulation is as follows: uniformly pick a position (i, j) of L and pick a neighbor position occupied by a cell c (uniformly distributed on the set $\mathcal{N}(i, j)$), i.e. the candidate flip. The CPM probabilistically accepts or rejects the flip, i.e. keeps L or update it to $L[c \leftarrow (i, j)]$, according to the energy of both lattices and the temperature-dependent probability distribution discussed in the main text, when $k_B T > 0$. Instead, when $k_B T = 0$ the distribution is

$$\mathcal{P}\Big(c \leftarrow (i, j)\Big) = \begin{cases} 0 & \text{if} \quad \Delta\mathcal{H} > 0 \\ \frac{1}{2} & \text{if} \quad \Delta\mathcal{H} = 0 \\ 1 & \text{if} \quad \Delta\mathcal{H} < 0 \end{cases}. \tag{15}$$

A computation starting at time t_s and ending at time t_e performs $t_e - t_s$ Monte Carlo steps each one attempting nmk random flips, as discussed in the main text. Once all the attempts of flips are finished, the new lattice is determined as a result of all the accepted flips. A stochastic process $\{L(t) \mid t = 0, 1, \ldots\}$ whose states are the lattice configurations, underlies the CPM. Technically, such a process is a Discrete Time Markov Chain.

The boolean model of Gene Regulatory Network

Let $\mathbf{x}(t)$ be the RBN binary vector-state at time t, its i-th component in $\mathbf{x}(t+1)$ is

$$\mathbf{x}(t+1)_i = f_i(\mathbf{x}(t)). \tag{16}$$

An execution of an RBN is a series of steps from an initial state $\mathbf{x}(0)$

$$\mathbf{x}(0) \to \mathbf{x}(1) \to \ldots \to \mathbf{x}(k_1) \to \mathbf{x}(k_2) \to \ldots \to \mathbf{x}(k_n) \to \mathbf{x}(k_1) \to \ldots$$

which, since the state-space is finite (i.e. for w genes there exist at most 2^w vectors in $\{0, 1\}^w$) and the dynamics is fully deterministic, will enter a limit cycle $\mathbf{x}(k_1) \to \ldots \to \mathbf{x}(k_n)$ from any initial condition. Such a cycle is termed *RBN attractor*, the sub-sequence from $\mathbf{x}(0)$ to $\mathbf{x}(k_1)$ (excluded) is its *transient*; the set of initial conditions ending up to the same attractor is its *basin of attraction*.

Our approach first selects, either exhaustively or randomly, a set $I = \{\mathbf{x} \in \{0, 1\}^w\}$ of initial conditions, and determines numerically its associated set of attractors A_I. Iteratively, in each attractor in A_I random flips are performed, i.e. some $\mathbf{x}_i$ is complemented in $\{0, 1\}$ yielding a new state $\mathbf{x}'$. The attractor α reachable from $\mathbf{x}'$ is then computed and included in A_I, if $\alpha \notin A_I$. By an arbitrary iteration of this process the ATN $\mathcal{A}$ of the subsumed NRBN is drawn as follows: for each perturbation yielding a

jump among attractors α and β the entry $a_{\alpha,\beta} \in \mathcal{A}$ is incremented, the matrix is finally normalized. As running example, consider the following ATN

$$\left(\begin{array}{c|cccc} \mathcal{A} & \alpha_1 & \alpha_2 & \alpha_3 & \alpha_4 \\ \hline \alpha_1 & 0.886 & 0.064 & 0.001 & 0.049 \\ \alpha_2 & 0.068 & 0.877 & 0.054 & 0.001 \\ \alpha_3 & 0 & 0.06 & 0.896 & 0.044 \\ \alpha_4 & 0.053 & 0.004 & 0.052 & 0.891 \end{array}\right) .$$

The differentiation tree T induced by $\mathcal{A}$ is generated according to the strategy discussed in [30]. With no aim of exhaustivity we briefly recall it here. Firstly, $\mathcal{A}$ is interpreted as the *adjacency matrix* of the graph of the attractors $G_{\mathcal{A}}$. Secondly, the set of *strongly connected component* in $G_{\mathcal{A}}$ is evaluated by standard algorithmic techniques; this is the TES at threshold 0, T_0, which is supposed to contain all the nodes of $G_{\mathcal{A}}$. If T_0 does not contain all the required nodes, the RBN is rejected and the process restarts with a new network. Otherwise, T_0 is assigned to the root of the differentiation tree. In the above ATN, $T_0 = \{(\alpha_1, \alpha_2, \alpha_3, \alpha_4)\}$.

This process now iterates proportionally to the size of the tree we want to generate, which has a maximum theoretical depth proportional to the number of nodes when T reduces to a path. The idea is that a threshold $0 < \delta < 1$ is picked, $\mathcal{A}$ is pruned of any $a_{\alpha,\beta} < \delta$, and the new number of TESs at threshold δ, T_δ, is evaluated. The set of direct descendants of the root of T is defined by T_δ via an injective map, i.e. each TES in T_δ defines a unique descendant. In the example above, when $\delta = 0.053$ the pruned ATN is (in bold the pruned entries)

$$\left(\begin{array}{c|cccc} \mathcal{A} & \alpha_1 & \alpha_2 & \alpha_3 & \alpha_4 \\ \hline \alpha_1 & 0.886 & 0.064 & \mathbf{0} & \mathbf{0} \\ \alpha_2 & 0.068 & 0.877 & 0.054 & \mathbf{0} \\ \alpha_3 & 0 & \mathbf{0} & 0.896 & \mathbf{0} \\ \alpha_4 & \mathbf{0} & \mathbf{0} & \mathbf{0} & 0.891 \end{array}\right)$$

which induces $T_0 = \{(\alpha_1, \alpha_2), (\alpha_3), (\alpha_4)\}$. The process stops when no more TESs emerge, and the last descendants determined become the leafs of T. For the example above further candidates thresholds are 0.06 and 0.068.

The multiscale link

Each $\mathcal{A}$, re-normalized once a threshold is fixed, is actually a Discrete-Time Markov Chain (DTMC) and the sub-matrix $\mathcal{A}_\theta$ associated to the emerging TESs θ is, by definition, a ergodic DTMC (i.e. it is possible to go from each state, in any number of steps, to every other state). In DTMCs terminology the transition probability matrix $\mathcal{A}_\theta$ is *irreducible* and thus the *stationary probability distribution* of the chain

$$\pi = \lim_{i \to \infty} \mathcal{A}_\theta^{(i)}$$

is unique and can be evaluated as the fixed point of the recursive transformation

$$\mathcal{A}_\theta^{(0)} = \mathbf{p}_0 \qquad \qquad \mathcal{A}_\theta^{(i+1)} = \mathcal{A}_\theta \mathcal{A}_\theta^{(i)} , \tag{17}$$

where $\mathbf{p}_0$ is an arbitrary initial distribution over the states of the chain. By fixing an arbitrary small ϵ, this fixed point can be evaluated yielding an approximation $\pi^* \approx \pi$, which can be used as explained in the main text.

Algorithmic implementation of the model

We describe the pseudocode of the model implementation. For clarity, we separate the general simulation driver for the model (Algorithm 1), the search of the suitable NRBN (Algorithm 2), the set up of the multiscale link (Algorithm 3), the external CPM dynamics (Algorithm 4) and the internal (Algorithm 5) dynamics.

Algorithm 1 High-level simulation driver.

Input: initial and final times (t_0 and T, in MCS), initial lattice L_0
NRBN-search(); {*search for suitable NRBNs, Algorithm 2*};
Multiscale-link(); {*set up the multiscale link, Algorithm 3*};
set $L \leftarrow L_0$ and $t \leftarrow t_0$; {*initialize time and the lattice*};
set t_N 0; {*this counts time cyclically, in NRBN time units*};
while $t < T$ **do**
  CPM-dynamics(); {*perform a single MCS of the CPM, Algorithm 4*};
  $t \leftarrow t + 1$; {*increase time in MCS steps*};
  if $t_N = 150/\hat{\ell}_\tau$ **then**
    NRBN-dynamics(); {*NRBN steps performed every* $150/\hat{\ell}_\tau$ *MCS, Algorithm 5*};
    $t_N \leftarrow 0$ {*reset counter*};
  else
    $t_N \leftarrow t_N + 1$; {*Increase time in NRBN steps*};
  end if
end while
Output: the final lattice L;

Algorithm 2 Noisy Random Boolean Network search with sampling.

Input: the NRBN parameters in Table 2, a lineage-commitment tree T;
repeat
  generate a n-nodes graph; {*uses, e.g., Erdös-Rènyi or Barabási-Albert algorithms*};
  assign boolean functions; {*functions can be, e.g., logical connectives, random or canalizing*};
  determine a set $I \subseteq \{0,1\}^n$ of initial conditions for the network; {*exhaustive or sampled*};
  for all $\mathbf{x}_0 \in I$ **do**
    determine the attractor reachable from $\mathbf{x}_0$; {*see equation (4)*};
  end for
  collect all the attractors to define the Attractors Transition Network (ATN);
  loop
    flip a node in an attractor state, determine the reachable attractor, update the ATN; {*exhaustive or sampled, according to the size of the state space*};
  end loop
  given all flips, set the probability of switching among attractors;
  determine the thresholds, the Threshold Ergodic Sets (TESs) and the NRBN-induced differentiation tree T_{NRBN} with the algorithm discussed in [30];
until T matches T_{NRBN} {*use a standard tree-matching algorithm*}
Output: the matched NRBN, the ATN, the TESs and the thresholds;

Algorithm 3 Multiscale link set up.

Input: the ATN and the TESs;
given the TESs, determine the steady state probability π; $\{$*use numerical algorithms for eq. (17)*$\}$;
given π, determine for each cell type τ the cycle length ℓ_τ as of equation (5);
determine the average cell cycle length $\hat{\ell}_\tau$;
evaluate the time unit conversion as of equation (6);
Output: each value ℓ_τ and $\hat{\ell}_\tau$;

Algorithm 4 Cellular Potts Model dynamics, single MonteCarlo step.

Input: the current lattice L;
for $h \cdot w \cdot k$ times (see Table 2) **do**
  pick a random lattice site l; $\{$*uniform distribution over* $L\}$;
  pick a random neighbor lattice site $l' \in \mathcal{N}(l)$; $\{$*uniform distribution over* $\mathcal{N}(l)\}$;
  evaluate the system energy $\mathcal{H}(L)$ as of equation (3);
  attempt the flip to l' according to equation (2), in case the flip is accepted update the lattice;
end for
Output: the resulting lattice;

Algorithm 5 Noisy Random Boolean Network, single time step.

Input: the ATN, the TESs and the lineage-commitment tree T;;
for all cells c in the lattice **do**
  let α be the attractor the cell is assigned to;
  by interpreting the TES and the corresponding ATN assigned to the current type of c as a Discrete-Time Markov Chain, perform a jump from α; $\{$*roaming models stochastic differentiation*$\}$;
  if c is Paneth and its apoptosis is triggered **then**
    kill c; $\{$*remind that the other cell types are shed through the lumen*$\}$;
  end if
  if c has completed its cell cycle **then**
    evaluate the next differentiation type τ according to the attractor the cell is assigned to, and T;
    divide the cell pixels $\mathcal{C}(c)$; $\{$*cell is doubled, this is mitosis*$\}$;
    assign to the newborn cells the type τ and reset their area target, if c is a stem cell one newborn is c itself; $\{$*force asymmetric division of stem cells*$\}$;
  else
    increase linearly the area target $A(c)$; $\{$*this is as of equation (7)*$\}$;
  end if
end for
Output: the new internal state of each cell;

Networks analysis

In our model GRNs drive the spatial dynamics, thus we analyzed different properties of the networks matching the crypt lineage tree. In Table 1 one can find some statistics for the 7 suitable network used in the simulations: (i) the length of any single attractor of the network (i.e. the number of states of all the gene activation patterns) $|\alpha|$; (ii) the robustness of any attractor, in terms of probability of remaining in the same attractor after one single flip perturbation, which is the first entry of ATN matrix, $\mathcal{A}(\alpha, \alpha)$; (iii) the stationary distribution of the ATN for each attractor $\pi(\alpha)$, which accounts for the average portion

of time (in percentage) spent in each attractor and which is used to weigh the length of attractors when computing the cell cycle length.

Please refer to the Results section for the comments on these measures.

Table 1. Properties of the suitable NRBNs used in the simulations.

$\mathbf{Net_1}$	α_1	α_2	α_3	α_4
$\lvert\alpha\rvert$	4	6	8	8
$\mathcal{A}(\alpha,\alpha)$	0.985	0.956	0.812	0.825
$\pi(\alpha)$	0.456	0.351	0.102	0.09

$\mathbf{Net_2}$	α_1	α_2	α_3	α_4	α_5
$\lvert\alpha\rvert$	7	1	7	7	7
$\mathcal{A}(\alpha,\alpha)$	0.945	0.98	0.882	0.914	0.894
$\pi(\alpha)$	0.325	0.083	0.117	0.09	0.383

$\mathbf{Net_3}$	α_1	α_2	α_3	α_4
$\lvert\alpha\rvert$	4	4	2	14
$\mathcal{A}(\alpha,\alpha)$	0.775	0.78	0.9	0.78
$\pi(\alpha)$	0.386	0.332	0.053	0.228

$\mathbf{Net_4}$	α_1	α_2	α_3	α_4
$\lvert\alpha\rvert$	1	1	1	1
$\mathcal{A}(\alpha,\alpha)$	0.8	0.74	0.92	0.9
$\pi(\alpha)$	0.371	0.342	0.057	0.228

$\mathbf{Net_5}$	α_1	α_2	α_3	α_4
$\lvert\alpha\rvert$	20	20	20	20
$\mathcal{A}(\alpha,\alpha)$	0.886	0.877	0.896	0.891
$\pi(\alpha)$	0.262	0.266	0.251	0.221

$\mathbf{Net_6}$	α_1	α_2	α_3	α_4
$\lvert\alpha\rvert$	8	13	4	7
$\mathcal{A}(\alpha,\alpha)$	0.732	0.821	0.975	0.791
$\pi(\alpha)$	0.245	0.176	0.358	0.219

$\mathbf{Net_7}$	α_1	α_2	α_3	α_4
$\lvert\alpha\rvert$	6	6	2	2
$\mathcal{A}(\alpha,\alpha)$	0.793	0.856	0.87	0.86
$\pi(\alpha)$	0.26	0.327	0.199	0.213
